# Transcription Activator-Like Effector Nucleases (TALEN)-Mediated Targeted DNA Insertion in Potato Plants

**DOI:** 10.3389/fpls.2016.01572

**Published:** 2016-10-25

**Authors:** Adrienne Forsyth, Troy Weeks, Craig Richael, Hui Duan

**Affiliations:** Simplot Plant Sciences, J.R. Simplot Company, BoiseID, USA

**Keywords:** TALEN, gene targeting, potato, double stand break, transgenic, homologous recombination

## Abstract

Targeted DNA integration into known locations in the genome has potential advantages over the random insertional events typically achieved using conventional means of genetic modification. Specifically integrated transgenes are guaranteed to co-segregate, and expression level is more predictable, which makes downstream characterization and line selection more manageable. Because the site of DNA integration is known, the steps to deregulation of transgenic crops may be simplified. Here we describe a method that combines transcription activator-like effector nuclease (TALEN)-mediated induction of double strand breaks (DSBs) and non-autonomous marker selection to insert a transgene into a pre-selected, transcriptionally active region in the potato genome. In our experiment, TALEN was designed to create a DSB in the genome sequence following an endogenous constitutive promoter. A cytokinin vector was utilized for TALENs expression and prevention of stable integration of the nucleases. The donor vector contained a gene of interest cassette and a promoter-less plant-derived herbicide resistant gene positioned near the T-DNA left border which was used to select desired transgenic events. Our results indicated that TALEN induced T-DNA integration occurred with high frequency and resulting events have consistent expression of the gene of interest. Interestingly, it was found that, in most lines integration took place through one sided homology directed repair despite the minimal homologous sequence at the right border. An efficient transient assay for TALEN activity verification is also described.

## Introduction

Plants can be improved for cropping purposes by inserting specific DNA sequences into their genomes. The DNA sequences are comprised of sequences that, when expressed in plants, confer a new trait to plants. Various methods have been developed for plant transformation that offer no control over the site into which introduced DNA sequences eventually reside in the genome. Such random insertions present several problems. Insertion can occur either in a transcriptionally active or inert region that may affect the expression level of the transgene (Peach and Velten, 1991). A transgene or a portion of DNA sequence could be inserted within a native gene sequence and consequently abolish the function of that gene. It is also possible to unintentionally create a new open reading frame at the junction of the insert and native DNA, which when expressed could negatively affect the quality or agronomic characteristics of the transgenic plant. Thus, given the unpredictable aspects of random insertion through transformation, introduction of a new trait into a crop requires generating a large number of transgenic lines. Subsequent analyses are also complicated due to the differences between all independent transgenic lines. Especially for vegetatively propagated crops like potato, trait introgression or insert elimination through crossing is complicated. The broad introduction of transgenic traits into asexually propagated crops would require genetic engineering of many commercially important varieties. Given the large number of transgenic lines needed to be produced in each variety, it is very difficult to justify financially.

For many reasons, targeted transgene integration has long been sought after in plant genetic modification. Original efforts utilized large stretches of sequence with homology to host target DNA to induce homologous recombination (HR), but very low targeted integration frequencies were achieved (Kempin et al., 1997; Hanin and Paszkowski, 2003). Subsequently, positive-negative selection methods were used to increase the efficiency of HR-mediated targeted integration in rice and *Arabidopsis* (Wang et al., 2001; Terada et al., 2002, 2007; Lida and Terada, 2005). The efficiency of HR based knock-in is also enhanced by modifying or expressing the components in the HR pathway, such as helicase *RecQ*, resolvase *ruvC* and *Rec8* (Shalev et al., 1999; Reiss et al., 2000; Li et al., 2004). Another approach of targeted integration utilizes site-specific recombinase-mediated integration systems (Fukushige and Sauer, 1992; Albert et al., 1995; Golic et al., 1997; Day et al., 2000). In this approach, DNA is inserted into a previously introduced recombination site in the genome which serves as a target. This is especially valuable for gene stacking when multiple site-specific recombination systems are combined. The newly integrated molecule contains a second recombination site that will serve as the target for the next round of DNA integration (Ow, 2011; De Paepe et al., 2013). Another approach was based on the observation that induced double strand breaks (DSBs) could facilitate targeted T-DNA integration in the plant genome (Puchta et al., 1993; Chilton and Que, 2003; Tzfira et al., 2003). In these studies, a specific rare endonuclease site, such as I-SceI was first randomly introduced into the plant genome and subsequently used as target site to generate a DSB. However, generating a DSB in a specific desired target locus still remains a challenge.

Endonuclease-based genome editing enzymes such as, meganucleases (Epinat et al., 2003), Zinc finger nucleases (ZFN) (Porteus and Baltimore, 2003), Transcription activator-like effector nucleases (TALEN) (Bogdanove and Voytas, 2011; Li et al., 2011) and CRISPR-associated (Cas) endonucleases (Jinek et al., 2012; Mussolino and Cathomen, 2013; Xie and Yang, 2013; Bortesi and Fischer, 2015) provide tremendous possibilities for generating DSBs in a targeted DNA region. Once this DSB occurs, the plant DNA repair machinery may either repair the break through a non-homologous end joining (NHEJ) pathway, which is imprecise and creates mutations to achieve gene knock out, or through a HR pathway to achieve gene replacement or insertion (Symington and Gautier, 2011). Several reports have shown the usage of these nucleases for targeted DNA integration in various plant species. These include: ZFN: Wright et al. (2005), Cai et al. (2009), Shukla et al. (2009), Townsend et al. (2009), Ainley et al. (2013), and De Pater et al. (2013); Talen: Zhang et al. (2013); Meganucleases: Puchta et al. (1993), Chilton and Que (2003), D’Halluin et al. (2008), D’Halluin et al. (2013) and Cas9: Li et al. (2013), Shan et al. (2013), and Svitashev et al. (2015).

In the present study, we describe a two-plasmid, TALEN-based strategy to induce a DSB in a preselected location of the potato genome and integrate a gene of interest cassette into this site. More specifically, the first plasmid contains a pair of TALENs designed to induce DSB in the intron of the 5′ UTR of a constitutively expressed *Ubiquitin7 (Ubi7)* gene. The plasmid was designed to express TALENs transiently. If stable integration of the TALEN expression cassettes occurs, it will be negatively selected out. Targeted insertion of T-DNA is achieved when a second donor plasmid comprised of a promoter-less mutated herbicide resistant gene (non-autonomous marker) and a gene of interest cassette are co-transformed into the potato plant. Herbicide resistant lines will develop only if the T-DNA is inserted into the targeted region and the transformants confer herbicide resistance. Herbicide resistant transformants were analyzed and our results indicated a high efficiency of targeted T-DNA integration. Although the original strategy of integration was based on NHEJ mechanism, it was discovered that the minimal homologous sequence at the right border resulted in one-sided HR-mediated integration.

## Materials and Methods

### Potato Transformation

Ranger Russet potato (*Solanum tuberosum cv Ranger Russet*) tissue culture stock plants were maintained in magenta boxes containing 40 ml half-strength Murashige and Skoog (MS) medium (Murashige and Skoog, 1962) modified by replacing ferrous sulfate and disodium EDTA with 36.7 g/l of ferric sodium EDTA, 30 g/l sucrose and 2 g/L gelzan. All plants were maintained in a growth chamber at 24°C under a 16 h photoperiod. Plants were inoculated with a mixture of *Agrobacterium* containing pSIM2170 and pSIM2370 constructs. The explants were transferred onto co-culture medium containing one-tenth MS modified basal medium with Gamborg’s vitamins (Gamborg, 1966), 30 g/l sucrose and 6 g/l agar on filter paper, under low light conditions. Explants were transferred and remained on callus induction medium (CIM) containing MS modified basal medium with Gamborg’s vitamins, 2.5 mg/l zeatin riboside, 0.1 mg/l napthaline acetic acid, 30 g/l sucrose, 300 mg/l timentin, 1.2 ml/l plant preservative mixture (PPM) and 6 g/l agar. After 2 weeks, explants were transferred to CIM containing 2.0 mg/l imazamox for selection and maintained for 4 weeks, transferring to fresh medium every 2 weeks. Explants were then transferred to shoot induction medium (SIM) containing MS modified basal medium with Gamborg’s vitamins, 2.5 mg/l zeatin riboside, 0.3 mg/l giberellic acid, 30 g/l sucrose, 2.0 mg/l imazamox, 300 mg/l timentin, 1.2 ml/l PPM and 6 g/l agar. Explants were cultured on SIM for 6 weeks and transferred every 2 weeks to fresh medium. Shoots were recovered for 2 weeks and then were rooted on one-half strength MS modified basal medium with Gamborg’s vitamins containing 300 mg/l timentin, 1.2 ml/l PPM and 0.5 mg/l imazamox.

### Construct Preparation

Transcription activator-like effector nuclease backbone originated from Hax3 TALE from Brassicaceae pathogen *X. campestris* pv. *Armoraciae* strain, a member of the AvrBs3 family. Plant codon optimized Hax3 sequence (Mahfouz et al., 2011) was synthesized by Invitrogen (Thermo Scientific, Waltham, MA USA) as three fragments: N terminal with a SV40 nuclear localization sequence (NLS), repeat variable diresidue (RVD), and truncated C-terminal with a FokI cleavage domain (Kim et al., 1997). The N-terminal and C-terminal fragments were used universally for both expression cassettes. The RVD was specifically designed for each DNA target. For a pair of effector nucleases, two expression cassettes were constructed. The first cassette (forward) contained a figwort mosaic virus (FMV) promoter and octopine synthase (OCS) terminator. The second cassette (reverse) contained a 35S promoter and Nos terminator. Each TALEN expression cassette was assembled in the pBluescript SK (-) cloning vector (Agilent Technologies, Santa Clara, CA, USA) and both forward and reverse TALEN cassettes were assembled in a binary vector using conventional restriction enzyme cloning techniques.

The target plasmid, pSIM2167, used for testing transient TALEN efficiency was constructed by in-frame fusion of a 60 bp target DNA sequence, including forward and reverse TALE binding sequences and a spacer, to a GUS reporter gene driven by an FMV promoter in a binary vector. In the spacer region of the sequence, there is an in-frame stop codon, as well as an *AluI* enzyme site. The donor plasmid was constructed by placing a promoter-less mutated potato *ALS* gene (*mStALS*) at the right border side followed by the gene of interest cassette. In this proof of concept study, the donor plasmid pSIM2370 contains a plant antibiotic resistant gene (*nptII*) expression cassette.

### Transient Infiltration

A transient infiltration assay in *Nicotiana benthamiana* leaves was used to test TALEN function and activity. Wild type *N. benthamiana* plants were grown for *Agrobacterium* infiltration experiments and maintained under standard greenhouse conditions with an ambient temperature of 22–25°C and 16 h light period. For assays, mature leaves of 4–6 weeks old plants were used.

Two binary vectors were utilized in this assay, pSIM2167 provided the target sequence for the TALEN and pSIM2170 contained the pair of TALENs. Both binary vectors were transformed into *Agrobacterium* strain AGL1and the resulted *Agrobacterium* were used for infiltration assays. *N. benthamiana* leaves were co-infiltrated with a mix of pSIM2167 + pSIM2170 *Agrobacterium* cultures, as well as either pSIM2167 or pSIM2170 alone as negative controls into two zones on opposite leaf halves. Prior to infiltration a suspension of *Agrobacterium* harboring the target and TALENs were suspended in a 1:1 ratio which were co-infiltrated at a combined OD600 of 0.4 or independently at an OD600 of 0.3. A 1ml syringe was used to deliver *Agrobacterium* into the abaxial side of third and fourth leaves from top.

### Gus Staining

Transient GUS assays were performed to visualize TALEN activity in the co-infiltrated leaves. Three days post inoculation, two leaf disks of approximately 0.9 cm were collected. The leaf disks were subsequently incubated in X-Gluc (5-bormo4-choror3-indolyl-betd glucuronide) staining solution, briefly vacuum infiltrated, incubated at 37^o^C overnight, and destained in 70% EtoH over a 2 days period. The leaves were then visualized under the microscope. Experiments were performed twice with similar results.

### Sequence Analysis

To detect modifications in the target sequence of the infiltrated plants, 5 mm leaf disks were excised from the infiltrated leaf regions 2–3 days post infiltration. Genomic DNA was extracted using a CTAB based method (Porebski et al., 1997). Approximately 100 ng of DNA was digested with *AluI* enzyme. Amplification was conducted with 20 ng of the digested DNA in a PCR reaction containing 0.5 mM primers HD184F and HD184R, which spans 200 bp on each side of the targeted region. The resulted PCR fragment was subsequently digested with *AluI* enzyme for the enrichment of the mutated target sequence. Undigested fragment (loss of *AluI* site) was cloned and multiple colonies were sent for sequence analysis for each of the treatment groups.

### PCR Screening and Genotyping

Insertion specific primer pair HD175F and HD198R was designed and used for PCR genotyping for TALEN-mediated targeted insertion (**Supplementary Table S1**). DNA was isolated from tissue culture leaf material using a CTAB method (Porebski et al., 1997). DNA underwent PCR amplification and products were cloned and sequenced.

### Southern Blot

Non-radioactive digoxigenin genomic Southern blotting was used to detect targeted DNA insertion and copy number. Plants were grown in the GH under conditions previously described. Genomic DNA was isolated via standard CTAB method from 0.5 g of young leaf tissue (Porebski et al., 1997). Six microgram of genomic DNA was digested with *HindIII* enzyme. The digested DNA was run on a 0.8% agarose gel in TBE buffer overnight. The gels were denatured and transferred to Hybond-N+ membrane (GE Healthcare Life Science, Pittsburg PA USA). A 1.1 kb DIG labeled DNA probe for *StALS* was synthesized by PCR amplification with the primer pair HD230F and HD230R (**Supplemental Table S1**) according to the manufacturer’s protocol (Roche Diagnostic, Indianapolis, IN, USA). The DNA probe was heat denatured and added to the Roche DIG easy Hyb buffer and rotated at 42°C overnight. Washing and developing was carried out via manufacturer’s instructions (Roche Diagnostic, Indianapolis, IN, USA). After exposure, the film was developed using a Konica SRX-101A developing machine.

### Northern Blot

RNA from field tissue was isolated using Tri Reagent according to the manufacturer’s protocol (Sigma–Aldrich, St. Louis, MO, USA). Fifteen microgram of RNA was run on a standard 1% agarose gel in MOPS. The gels were washed with 10xSSC and blotted to Hybond-N+ membrane (GE Healthcare Life Science, Pittsburg, PA, USA). An *nptII* DIG labeled probe was synthesized with primers HD288F and HD288R to produce a 0.5 kb probe according to the manufacturer’s protocol (Roche Diagnostic, Indianapolis, IN, USA). The DNA probe was heat denatured and added to the Roche DIG easy Hyb buffer and rotated at 42°C overnight. Washing and developing was carried out via manufacturer’s instructions (Roche Diagnostic, Indianapolis, IN, USA). After exposure, the film was developed using a Konica SRX-101A developing machine.

## Results

### Strategy of Site Specific Integration, Design of Insertion Site and TALENs

**FIGURE 1:**
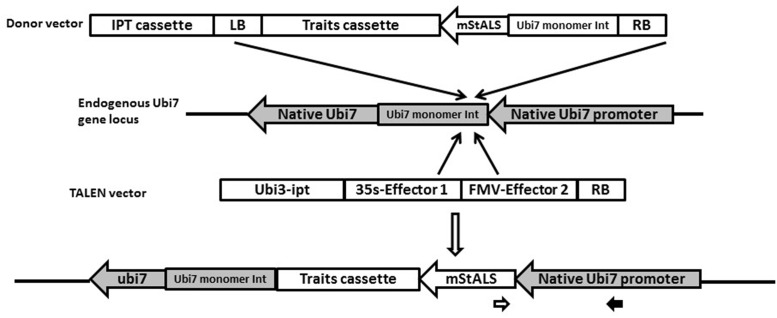
**Schematic of strategy for the targeted T-DNA insertion.** The TALEN vector contains a pair of TALENs targeting the endogenous potato Ubi7 intron immediately 5′ to the initiation codon for the first ubiquitin monomer. The LB was removed and an *ipt* overexpression cassette was put next to the TALEN expression cassettes on the left side of T-DNA. The donor vector contains a promoter-less mutated potato *ALS* gene (*mStALS*) next to the RB and the *nptII* cassette. Upon *Agrobacterium*-mediated transformation, expression of TALENs generate DSB at the endogenous target, once the donor T-DNA is inserted into this endogenous site, the endogenous promoter will drive the expression of *mStALS* expression and resulted targeted events can be selected out on herbicide containing medium. The targeted events can also be PCR confirmed by using a primer in the *mStALS* gene (opened arrow) paired with a primer within the endogenous Ubi7 promoter (solid arrow).

Transgene expression varies greatly due to position effects of random insertion in transgenic plants. Targeted insertion of a transgene into a specific and active location of the chromosome is desirable for both line selection and downstream characterization. Our approach used TAL effector nucleases to introduce targeted DSB in the potato genome immediately following an endogenous promoter, so that the provided cassette harboring a native promoter-less herbicide resistant marker and transgene of interest can be captured in that site. The target site chosen for this study was the intron region within the 5′ UTR of the potato *Ubi7* gene. As shown in **Figure 1**, once inserted into the desired site, the endogenous *Ubi7* promoter will drive the expression of the promoter-less marker gene which will then confer herbicide resistance and enable the regeneration of transgenic plants on medium containing the appropriate selective agent. For this study, the marker gene used was the mutated acetolactate synthase gene *(ALS)* (also known as acetohydroxy acid synthase, or *AHAS*). ALS catalyzes the first step in the synthesis of the branched-chain amino acids and is sensitive to a number of herbicides such as imazamox. Amino acid substitutions provide tolerance to the herbicides in a number of species including potato (Andersson et al., 2003). We engineered two amino acid substitutions in the potato *StALS* gene, designated *mStALS*. The DNA and protein sequences with nucleotide and amino acid substitutions are shown in **Supplementary Figures S1** and **S2**.

We chose the potato *Ubi7* gene as our target site because it is constitutively expressed in potato plants and has a promoter that is commonly used for constitutive gene expression in potato transgenic research and applications (Richael et al., 2008). *Ubi7* transcripts have an intron in the 5′ UTR. The intron along with the first monomer have been shown to contribute to high expression levels of the transgene (Garbarino et al., 1995). Therefore to promote high expression of our transgene, we included the intron and first monomer in the design of the promoter-less herbicide resistant gene. A pair of TAL effector nucleases were designed to make a DSB within the intron, which was (a) >25-bp upstream from the branch site [consensus = CU(A/G)A(C/U)], pyrimidine-rich ( = AT-rich) region, and intron/exon junction (consensus = CAGG), and (b) > 50-bp downstream from the splice donor site at the exon/intron junction (consensus = AGGT) (Lou et al., 1993). The sequence of the target region is shown in **Supplementary Figure S3**. The TALEN scaffold used in our experiments is plant codon optimized Hax3 (Mahfouz et al., 2011), a member of the AvrBs3 family that was identified in *Brassicaceae* pathogen *Xanthamonas campestris pv. armoraciae* strain 5 (Kay et al., 2005). Other modifications made to the scaffold included: (a) truncation of the C-terminal activation domain of the original Hax3 (last 215 amino acids); (b) addition of a NLS from SV40 virus at the N terminal of the truncated Hax3 protein (Lassner et al., 1991). The DNA and protein sequences of the final forward and reverse TALENs can be found in the supplementary data (**Supplementary Figures S4**–**S7**).

**FIGURE 2:**
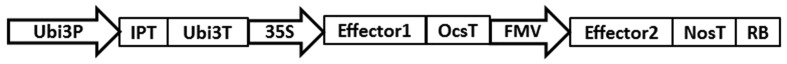
**Schematic of binary vector pSIM2170 that contains forward and reverse TALENs.** Two TALENs were driven by either 35s or FMV constitutive promoter followed by either OCS or Nos terminator, respectively. Each TALEN has a SV40 nuclear localization sequence (NLS), designed repeat variable diresidue (RVD) (E3 or E4 repeat), and truncated C-terminal fused to a FokI cleavage domain. A potato Ubi3 promoter-driven *ipt* gene cassette was put on the left side of T-DNA and the T-DNA LB was removed in this vector.

A binary vector was created for transient expression of the TALENs in plant cells. This vector contained two TALENs each operably linked to a strong constitutive promoter [Cauliflower Mosaic Virus (CaMV) 35s or Figwort Mosaic Virus] and followed by a terminator [nopaline synthase (Nos) or octopine synthase] to form two separate plant expression cassettes. The two cassettes were cloned into a pSIM binary vector to form pSIM2170 as shown in **Figure 2**. To facilitate transient expression of the effectors, the left border (LB) was removed from pSIM2170. A plant hormone cytokinin biosynthesis *isopentenyl transferase (ipt)* gene expression cassette was included at the left side of the TALEN cassettes. If stable transformation occurs, the entire vector sequence is integrated into the plant cell’s genome. The stable integration of the *ipt* expression cassette results in the overproduction of cytokinin in cells and shoots. These shoots are stunted and fail to develop roots (Richael et al., 2008), which allows for stably integrated TALEN sequences to be eliminated based on phenotype. This reduces the number of plants with stably integrated effector expression cassettes into the plant genome for further analysis.

### Validation of the Designed TALEN in Transient Expression System

**FIGURE 3:**
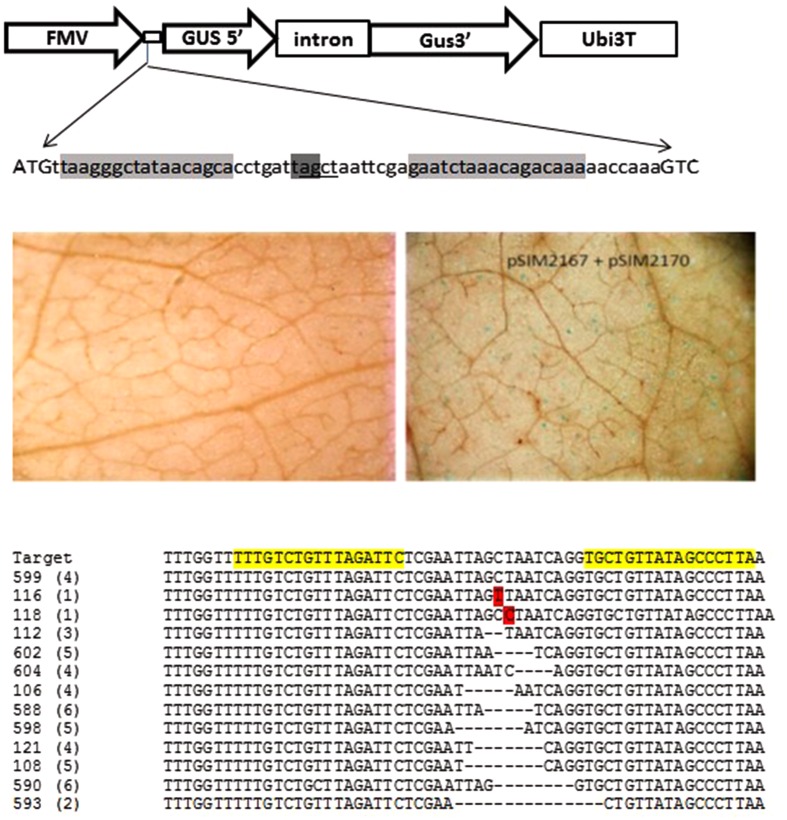
**Verification of designed TALENs in *N. benthamiana* infiltration.** **(Top panel)** Target vector pSIM2167 of TALENs for *N. benthamiana* infiltration. A 60 bp DNA sequence from *StUbi7* 5′ intron was inserted between the first and second amino acid codons of the *GUS* gene. There is an in frame stop codon (dark gray highlighted) in the spacer region between the two recognition sites (light gray highlighted) which will abolish the GUS protein translation. TALEN-mediated DNA cleavage and subsequent NHEJ based repairing will destroy the stop codon and translation of the GUS will be in frame and restore GUS activity. An *AluI* restriction enzyme site (underlined) was also included in the spacer to facilitate detection of TALEN-mediated indels. **(Middle and bottom panels):** The functionality of designed TALEN in transient assay. Target construct was *Agro*-infiltrated into *N. benthamiana* leaves alone or together with the TALEN construct. Forty-eight hours after infiltration, leaf tissue at the infiltrated site was collected and stained with GUS staining solution and de-stained with ethanol for microscopic examination. DNA was isolated from co-infiltrated tissue, digested with *AluI*, PCR amplified, cloned and sequenced. **(Middle left panel):** Target construct pSIM2167 alone. **(Middle right panel):** Target construct pSIM2167 co-infiltrated with TALEN construct pSIM2170. **(Bottom panel):** Sequences of target region with various modifications. The number in the parenthesis represents how many times the modification occurred in the 50 clones sequenced. The numbers on the left are identifiers of the respective clones.

To demonstrate the efficiency of the designed TALENs and their ability to recognize the target sequence and induce a DSB, we took advantage of a transient infiltration method in *N. benthamiana*. For this purpose, a target vector pSIM2167 was designed for co-infiltration into *N. benthamiana* with the TALEN construct previously described (pSIM2170). The target vector contains a modified β-glucuronidase (*GUS)* reporter gene driven by a constitutive FMV promoter. The *GUS* ATG start codon immediately precedes a 60 bp target sequence containing both forward and reverse recognitions sites. Between the two recognition sequences, there is a stop codon in frame with the *GUS* coding sequence which rendered this form of the *GUS* gene non-functional (**Figure 3**, Top panel). Binary vectors pSIM2170 and pSIM2167 were transformed into *Agrobacterium* strain AGL1 and subsequently co-infiltrated or infiltrated individually into *N. benthamiana* leaves. Infiltrated leaf disks were collected 2–3 days post infiltration and GUS staining was performed. Leaves infiltrated with pSIM2167 alone showed no GUS expression, demonstrating that the stop codon present in the spacer region led to the inactivation of GUS due to early termination of the coding sequence (**Figure 3**, Middle left panel). When leaves were co-infiltrated with the TALEN construct pSIM2170 and the target construct pSIM2167, the TALENs induced DSBs and cleaved the region containing the stop codon in the target sequence and were subsequently repaired by *N. benthamiana* cellular DNA repair NHEJ machinery. This in many cases resulted in the loss of the stop codon. When the repaired form of the target sequence was in frame with the downstream *GUS* coding sequence, GUS protein function was recovered and visualized by a standard GUS staining assay (Jefferson et al., 1987) (**Figure 3**, Middle right panel). GUS expression results indicated that the designed TALENs cleaved the stop codon in the target region and was successfully repaired by the cellular repair mechanism.

To further validate the functionality of the designed TAL effectors, sequence analysis was performed on leaves 3 days post infiltration. The target sequence region was PCR amplified and sequenced to identify TALEN mediated mutations. To accommodate for the unpredictable nature of the transient assay with the newly designed TALENs, a four nucleotide restriction enzyme site (*AluI*) was included between the two recognition sites. Direct PCR and cloning of the target sequence yielded large amounts of unmodified target sequence. The genomic DNA isolated from the infiltrated tissue was therefore digested with *AluI* enzyme prior to PCR amplification to reduce the quantity of unmodified target sequence. The PCR product underwent a second round of digestion with *AluI* enzyme to further enrich the modified target sequence prior to cloning and sequence analysis. Target site sequence analysis showed no modifications present in the tissue infiltrated with pSIM2167 alone (data not shown). However, individual clones obtained from the samples co-infiltrated with pSIM2170 and pSIM2167 shown in **Figure 3** Bottom panel confirmed TALEN mediated modifications in the target region. Of the 50 clones sequenced, 4 had unmodified target sequences, one contained a single base pair substitution, one contained a single base pair insertion, and 44 harbored deletions ranging from 2 to 15 bp (**Figure 3**, Bottom panel).

### Integration of Gene of Interest Cassette into Targeted Location in Potato Genome

**FIGURE 4:**
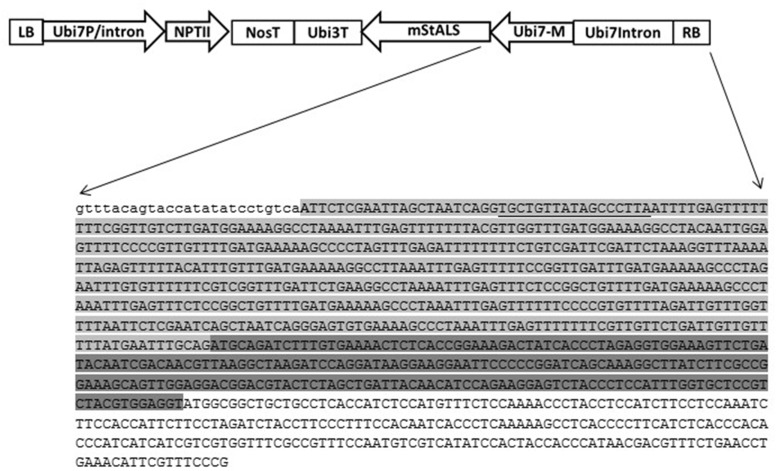
**Design of knock-in donor vector.** The donor vector pSIM2370 T-DNA contains a *StUbi7* promoter driven *nptII* gene cassette at the LB side, and a promoter-less m*StALS* gene at the RB side. At the sequence level, next to the RB, are sequences of a partial *Ubi7* 5′ intron region (light gray highlighted), *Ubi7* monomer (dark gray highlighted) fused to the *mStALS* coding sequence (only partial sequence is shown in this figure). The intron sequence contains a spacer between the two TALEN binding sites and the second TALEN recognition site (underlined). There is no *ipt* expression cassette on the backbone of donor vector.

**FIGURE 5:**
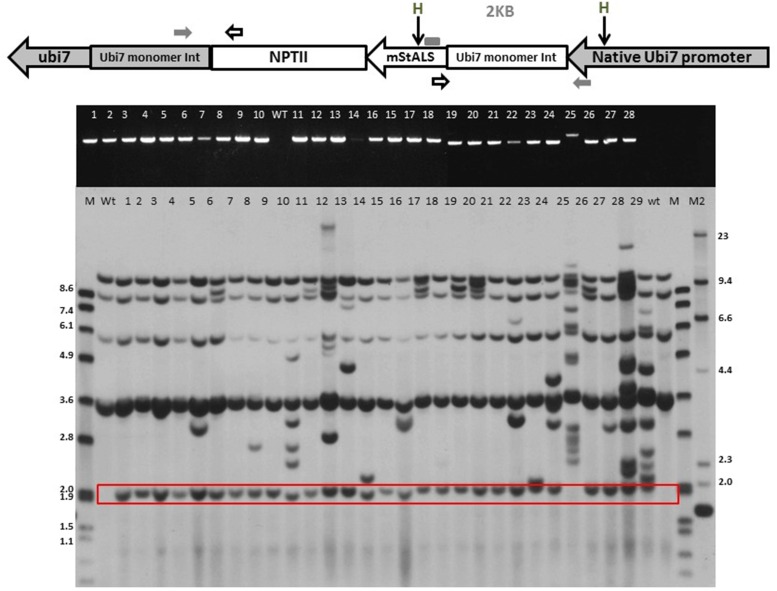
**Identification of targeted integration events.** DNA from herbicide resistant transgenic lines were isolated for PCR and Southern analyses. **(Top panel)** Schematic of the *Ubi7* locus after site-specific gene integration. Primers 1: within the endogenous Ubi7 promoter and 2: within the *mStALS* gene coding region are indicated by arrows. Two *HindIII* restriction enzyme sites, one in the endogenous *Ubi7* promoter and one in the *mStALS* coding region are also labeled. **(Middle panel)**: PCR using primers 1 and 2 detects site-specific integration events. **(Bottom panel)**: Southern blot using *StALS* probe detects the site-specific integration band at predicted size (boxed). The size of dig-labeled ladders were marked as kb (M: Dig-labeled marker 7, M2: Dig-labeled marker 2).

After functional confirmation of the designed TALENs in the *N. benthamiana* transient system, we tested the ability of the designed TALENs to facilitate targeted transgene integration of the provided insert cassette into the potato genome. We designed a donor vector, pSIM2370 comprised of two cassettes: the first cassette contains a promoter-less herbicide resistant gene, mutated potato acetolactate synthase (*mStALS*) gene, and the second consists of a selection marker (*nptII*) expression cassette (**Figure 4**). Following the right border, the sequence comprised of: part of the *StUbi7* gene 5′-UTR intron, a spacer sequence located between the two targeted TAL binding sites, a Ubi7 monomer-encoding sequence fused to *mStALS* gene that is insensitive to ALS inhibitor and a terminator of the potato *ubiquitin-3* gene. The 5′ sequence of this cassette is shown in **Figure 5**. The second plant antibiotic resistant gene (*nptII*) expression cassette included a *nos* terminator, an *nptII* coding region and a *Ubi7* promoter near the LB. A total of 900 Russet Ranger potato tissue culture explants were co-transformed with the TAL effector nuclease vector pSIM2170 and donor vector pSIM2370 as described in the experimental procedures. When the provided transgene was integrated into the targeted genome position, regenerated transgenic plants contained the promoter-less herbicide resistant gene driven by the endogenous *Ubi7* promoter. In our experiment, we generated a total of 63 independent herbicide resistant lines for further characterization.

### Characterization of Herbicide Resistant Transgenic Lines

**FIGURE 6:**
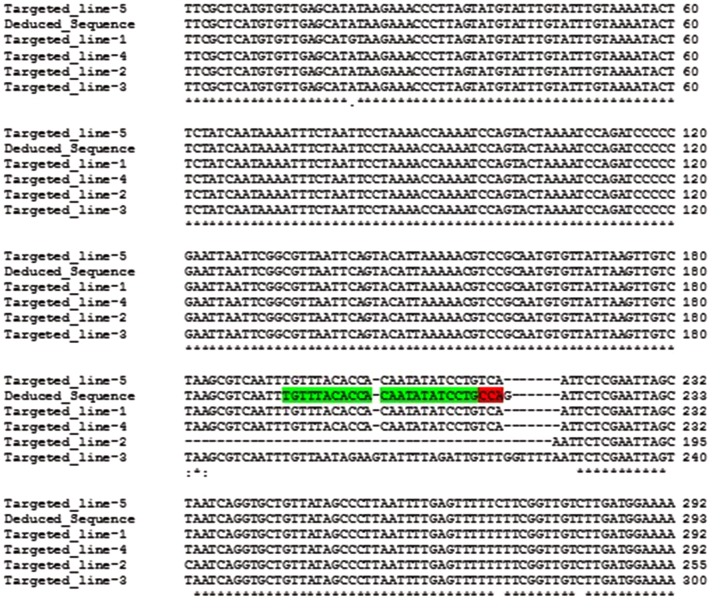
**Sequence of LB side flanking region.** A pair of primers, one within the endogenous *Ubi7* intron and the other within the T-DNA region of the donor vector close to the left border (as shown in **Figure 5**), were used to amplify targeted insertion in the LB junctions. Amplified products were cloned and sequenced. Sequences of five lines are shown here and aligned to deduce the LB junction after targeted integration. The LB sequence is green highlighted in the deduced sequence with the last three nucleotides red-highlighted, which are lost in the perfect LB cleavage events.

To further validate the integration of the T-DNA into the targeted location, the herbicide resistant plants were characterized through PCR sequence analysis. Primers were designed to span the integration region, with the forward primer located in the endogenous *Ubi7* promoter and the reverse in the m*StALS* transgene. Integration of the target gene generates a 1.1 kb product. Complications during integration such as DNA re-arrangements, extra fragment insertions or deletions changes the size of the product and were visualized on an agarose gel. As shown in **Figure 6** middle panel, among the first 28 herbicide resistant lines screened by targeted insertion specific primers, 27 had a PCR product at the expected size (96%). We cloned and sequenced 19 PCR products with the expected sizes as well as the larger PCR product from line 25. We found that the transgene integrated into the endogenous Ubi7 intron region without any additional nucleotides in all 19 lines (**Supplementary Figure S8**). Line No. 25 produced a larger PCR product and when sequenced was found to contain an insert of an additional segment of the *Ubi7* intron (**Supplementary Figure S8**).

Targeted integration in herbicide resistant plants was further confirmed by Southern blot analysis. The assay was performed using *HindIII* digested genomic DNA and probed with a *StALS* coding sequence. The *HindIII* site is located in the promoter region of *Ubi7* gene and in the *ALS* gene coding region (**Figure 5**, top panel). DNA from transgenic lines digested with *HindIII* and probed with the partial *StALS* sequence upstream of the *HindIII* site should produce a targeted event specific band (2 kb) on a Southern blot. As shown in **Figure 5** bottom panel, four bands were generated in the wild type control. These bands are signals from the endogenous *StALS* genes. There are additional bands in all PCR positive herbicide resistant lines at the expected 2 kb size for targeted insertion. Although every event had a targeted insertion, many also contained multiple copies as indicated by additional bands on the Southern blot. To select lines for further investigation, the data from the Southern blots were used to determine single copy lines as opposed to PCR products. Among the 12 selected single copy lines, an additional Southern blot was performed with a probe that targeted the TALEN expression cassette. We found that only one line contained a small piece of the expression cassette. This result validated that the negative selection method utilizing the *ipt* gene eliminated the majority of transgenic lines containing TALEN construct (**Supplementary Figure S9**).

Additionally, we investigated the integration at the LB junction. If the T-DNA was inserted into the designed genome location, the T-DNA LB flanking sequence would be the endogenous *Ubi7* gene intron and monomer. We performed PCR flanking the LB sequence in all twelve single copy lines with a primer pair designed to amplify this region. We were able to amplify a product from 5 lines. As shown in **Figure 6**, all products aligned to a deduced junction sequence with variations at the LB cutting site. Three lines appeared to have a predicted cut at the LB cleavage site (between the 3rd and 4th nucleotides from 3′ end of border sequence, Van Haaren et al., 1988) and was linked to the intron of the endogenous *Ubi7* gene with three base pair filler nucleotides. Two sequences had a small deletion at the LB, the first fused directly to endogenous *Ubi7* intron without filler DNA and the second fused to the endogenous *Ubi7* intron with short filler DNA.

## Discussion

In this study, we demonstrated the ability to use TALEN induced DSB to integrate a GOI cassette into a specific target site in the potato genome. To facilitate the selection of integrated events, we chose a well characterized endogenous potato *Ubi7* promoter to drive the expression of a promoter-less marker gene.

In theory, any designed transgene with a promoter-less marker gene could be inserted immediately following a constitutive promoter to allow expression of the marker gene and other gene(s) of interest within the same T-DNA. To select for targeted insertion, we used the plant-derived *mStALS* gene as the selection marker. By choosing a native marker, we are able to integrate genes in a cisgenic manner since our entire cassette could originate from sexually compatible species; e.g., late blight genes from wild potato species. Other native markers such as plant derived 5-enolpyruvylshikimate-3-phosphate synthase (*epsps)* gene can also be used as a selection marker or used as a marker for re-transformation of an additional gene(s) (Sidorov and Duncan, 2009). Although it is possible to achieve site specific integration and selection, non-specific integration can still occur. To circumvent this issue, integration specific PCR and selection of single copy lines were used to screen out the events with unintended non-specific insertions.

When gene editing tools are used to generate a DSB, gene replacement/insertion is generally thought to occur via the HR pathway, which requires flanking homologous arms for insertion. Salomon and Puchta (1998) have previously shown that DSBs serve as a target site for T-DNA integration when the breaks are generated by the restriction enzyme I-SceI. It has also been reported that T-DNA can be captured and integrated into a DSB site via NHEJ pathway (Chilton and Que, 2003 and Tzfira et al., 2003). We therefore hypothesized that when a DSB is generated at a desired genomic location, there will be an increased likelihood that the T-DNA will integrate into that site, thus reducing the need for lengthy homologous arms. Shortening the length of homologous arms decreases the complexity of the donor construct. In our design, we fused the *Ubi7* monomer and part of the 5′-UTR intron to the marker gene. This gave our insert partial homology to the endogenous target site at the right border (RB). The majority of the homologous sequences are after the designed cutting site which differs from the typical homologous arm design. Our results indicated the possibility of achieving site specific gene insertion to a target site without the use of long homologous arms. We also observed fusion of the endogenous *Ubi7* sequence to our insert without additional nucleotides. This suggested that the integration of the insert at the RB site occurred through HR (**Supplementary Figure S8**). When we investigated the LB junction, we observed imperfect integration between the LB sequence of our construct and the endogenous *Ubi7* gene. The LB junction was either perfectly cut or contained filler nucleotides or small deletions. This occurrence is similar to that observed in regular T-DNA insertion junctions (De Buck et al., 1999). This suggests that our insertion events occurred via one-sided (RB) HR. The LB was fused to endogenous sequence downstream of the DSB in a NHEJ manner. Similarly, Puchta et al. (1996) demonstrated *in planta*, HR restricted to one side of the DSB could happen which was described as the non-conservative one-sided invasion model. However, for the identified single copy lines, not every LB junction was identified by PCR using our current primers. This was most likely due to the occurrence of large deletions resulting from NHEJ that caused the loss of primer binding sites. To bypass this issue, primers to amplify a larger region could be used. There could also be unknown mechanisms involved in LB integration making it difficult to detect the junction. Nevertheless, our results showed that in several single copy targeted events, it is feasible to identify the LB junction region using simple PCR methods (Five representative lines in **Figure 6**). Together with the observation that we obtained many lines that have multiple copies of random T-DNA insertion suggest that there are opportunities to improve the design of our donor vector. Previous studies conducting precise targeting events in maize and cotton did not observe additional random integrations (D’Halluin et al., 2008, 2013). However, this was not the case in our experiments which could possibly be attributed to the use of a different plant species. Our next steps will focus on donor architectures that can reduce the non-specific random T-DNA integration and increase the ratio of precise integration at the LB side.

Current research typically utilizes genome editing enzymes through stable integration in the plant. The gene that encodes the genome editing enzyme can be subsequently segregated out in the following generation. However, when working with a vegetatively propagated species such as potato, segregating the next generation is not feasible. In this study, we used a negative selection based approach to ensure transient expression of the genome editing nucleases during the transformation process. To do this, we designed the T-DNA region of nucleases plasmid to contain a plant hormone cytokinin biosynthesis cassette (*ipt*) alongside the TALEN cassettes. Ideally, when the TALEN cassette integrates into the plant genome, the *ipt* cassette will also be integrated. This will result in a phenotypically distinguishable plant that can be screened visually and discarded. Continual modification of the genome can occur with the presence of stably integrated TALENs, however by eliminating lines with stable integration of TALEN, they are unable to continue modifying the genome. This suggests that the lines containing the modifications underwent alterations during the regeneration process, most likely before first cell division occurred. This method could also be adapted to other genome editing tools such as ZFN, meganucleases and CRISPR/Cas9 system. Especially for CRISP/Cas9, given the simplicity of the system, knock-in gene of interest(s) into different genomic location is simpler since the designing of new nucleases is not needed. In our lab, when CRISPR/Cas9 are designed to cleave the same target described in this paper, similar T-DNA integration results were achieved (unpublished data).

We hypothesized that among the independent lines, if targeted transgene insertion is achieved, the variability of transgene expression levels should be minimal. For this purpose, 12 single copy targeted events were selected for an expression study. Northern blot results showed that expression of the *nptII* gene, the trait gene in our POC donor vector, in targeted lines was relatively uniform (**Supplementary Figure S10**). This is consistent to other targeted studies that utilized site-specific recombinase such as *Cre*-lox, *FLP*-FRT and ZFN (Srivastava et al., 2004; Nandy and Srivastava, 2011; Kumar et al., 2015). Currently, we have designed new donor vectors which contains a trait gene of interest and trait genes in both targeted and random events will be compared. However, the targeted insertion events could generate some uncharacteristic phenotypes. This could be due to somaclonal variation that often occurs during the cell dedifferentiation process. It has been shown in the *Cre*-lox site specific recombinase system that identical site-specific transgene integration is able to produce alleles that are differentially silenced. This is attributed to factors like epigenetic modifications such as methylation (Day et al., 2000). For that reason, we do not expect all targeted events to have the same phenotype. On the other hand, provided a good integration site is chosen, fewer events will be required if targeted integration is achieved.

Since a functional *Ubi7* promoter is used in the donor vector to drive the *nptII* gene expression, we also tested the possibility that this promoter activates the promoter-less *mStALS* gene. The donor vector (pSIM2370) was transformed into potato plants alone using kanamycin as selection agent. All 50 kanamycin resistant lines obtained were also grown on imazamox containing medium, and only three lines (none were single copy) survived. The ratio of resistant lines falls within the range of a promoter trapping experiment in which the T-DNA is trapped by an endogenous promoter. This indicated that the Ubi7 promoter used to drive *nptII* gene expression did not activate the promoter-less *mStALS* gene in the same T-DNA.

In summary, we report a method that relies on the transient expression of TALEN to generate transgenic lines with site-specifically integrated T-DNA. Additionally, single copy events with easily determined flanking sequences and consistent transgene expression can aid in the ease of characterization. As a result, this could support transgenic crop improvement with more precision, thereby greatly reducing the number of independent lines to be generated and the workload of downstream characterization.

## Author Contributions

HD designed the work, HD and AF conducted the experiments, analyzed and interpreted data, TW performed plant transformation. HD and AF drafted manuscript, and HD, AF, CR, and TW reviewed, edited and approved manuscript.

## Conflict of Interest Statement

The authors declare that the research was conducted in the absence of any commercial or financial relationships that could be construed as a potential conflict of interest.
