# Supplementary material for: Transcription Activator-Like Effector Nucleases (TALEN)-Mediated Targeted DNA Insertion in Potato Plants
Source: Front Plant Sci. 2016 Oct 25;7:1572. doi: 10.3389/fpls.2016.01572 (PMC5078815; doi:10.3389/fpls.2016.01572)
Supplement: Supplementary file 1 [file Data_Sheet_1.DOCX]

**Supplemental material:**

**Fig. S1 Sequences of modified potato *ALS* gene**

>m*StALS* cDNA atggcggctgctgcctcaccatctccatgtttctccaaaaccctacctccatcttcctccaaatcttccaccattcttcctagatctaccttccctttccacaatcaccctcaaaaagcctcaccccttcatctcacccacacccatcatcatcgtcgtggtttcgccgtttccaatgtcgtcatatccactaccacccataacgacgtttctgaacctgaaacattcgtttcccgtttcgcccctgacgaacccagaaagggttgtgatgttcttgtggaggcacttgaaagggagggggttacggatgtatttgcgtacccaggaggtgcttctatggagattcatcaggctttgacacgttcgaatattattcgtaatgtgctgccacgtcatgagcaaggtggtgtgtttgctgcagagggttacgcacgggcgactgggttccctggtgtttgcattgctacctctggtccgggagctacgaatcttgttagtggtcttgcggatgctttgttggatagtattccgattgttgctattacgggtcaagtgccgaggaggatgattggtactgatgcgtttcaggaaacgcctattgttgaggtaacgagatctattacgaagcataattatcttgttatggatgtagaggatattcctagggttgttcgtgaagcgttttttctagcgaaatcgggacggcctgggccggttttgattgatgtacctaaggatattcagcaacaattggtgatacctaattgggatcagccaatgaggttgcctggttacatgtctagattacctaaattgcctaatgagatgcttttggaacaaattattaggctgatttcggagtcgaagaagcctgttttgtatgtgggtggtgggtgtttgcaatcaagtgaggagctgagacgatttgtggagcttacgggtattcctgtggcgagtactttgatgggtcttggagcttttccaactggggatgagctttcccttcaaatgttgggtatgcatgggactgtgtatgctaattatgctgtggatggtagtgatttgttgcttgcatttggggtgaggtttgatgatcgagttactggtaaattggaagcttttgctagccgagcgaaaattgtccacattgatattgattcggctgagattggaaagaacaagcaacctcatgtttccatttgtgcagatatcaagttggcattacagggtttgaattccatattggagggtaaagaaggtaagctgaagttggacttttctgcttggagacaggagttaacggaacagaaggtgaagtacccattgagttttaagacttttggtgaagccatccctccacaatatgctattcaggttcttgatgagttaactaacggaaatgccattattagtactggtgtggggcaacaccagatgtgggctgcccaatactataagtacaaaaagccacaccaatggttgacatctggtggattaggagcaatgggatttggtttgcctgctgcaataggtgcggctgttggaagaccgggtgagattgtggttgacattgatggtgacgggagttttatcatgaatgtgcaggagttagcaacaattaaggtggagaatctcccagttaagattatgttgctgaataatcaacacttgggaatggtggttcaactggaggatcgattctataaggctaacagagcacacacttacttgggtgatcctgctaatgaggaagagatcttccctaatatgttgaaattcgcagaggcttgtggcgtacctgctgcaagagtgtcacacagggatgatcttagagctgccattcaaaagatgttagacactcctgggccatacttgttggatgtgattgtacctcatcaggagcacgttctacctatgattcccattggcggtgctttcaaagatgtgatcacagagggtgatgggagacgttcatattga

Green highlighted G804A is a silent mutation to eliminate BstEII sites which benefited the cloning process. Yellow highlighted TG1687/1688CT and G1925T mutations generated two amino acid substitutions, W563L and S642I, which confers herbicide resistance.

**Fig. S2 Sequences of modified potato ALS protein**

>mStALS Amino acid

maaaaspspcfsktlppsssksstilprstfpfhnhpqkasplhlththhhrrgfavsnvvisttthndvsepetfvsrfapdeprkgcdvlvealeregvtdvfaypggasmeihqaltrsniirnvlprheqggvfaaegyaratgfpgvciatsgpgatnlvsgladalldsipivaitgqvprrmigtdafqetpivevtrsitkhnylvmdvediprvvreafflaksgrpgpvlidvpkdiqqqlvipnwdqpmrlpgymsrlpklpnemlleqiirliseskkpvlyvgggclqsseelrrfveltgipvastlmglgafptgdelslqmlgmhgtvyanyavdgsdlllafgvrfddrvtgkleafasrakivhididsaeigknkqphvsicadiklalqglnsilegkegklkldfsawrqelteqkvkyplsfktfgeaippqyaiqvldeltngnaiistgvgqhqmwaaqyykykkphqwltsgglgamgfglpaaigaavgrpgeivvdidgdgsfimnvqelatikvenlpvkimllnnqhlgmvvqledrfykanrahtylgdpaneeeifpnmlkfaeacgvpaarvshrddlraaiqkmldtpgpylldvivphqehvlpmipiggafkdvitegdgrrsy

Mutations W563L and S642I that confers the herbicide resistance are yellow highlighted.

**Fig. S3 Potato *Ubi7* gene 5’ intron and first monomer region**

CTATTATAATTTCTCTCAATTGCCTTCAAATTTCTCTTTAAGGTTAGAAATCTTCTCTATTTTTGGTTTTTGTCTGTTTAGATTCTCGAATTAGCTAATCAGGTGCTGTTATAGCCCTTAATTTTGAGTTTTTTTTCGGTTGTCTTGATGGAAAAGGCCTAAAATTTGAGTTTTTTTACGTTGGTTTGATGGAAAAGGCCTACAATTGGAGTTTTCCCCGTTGTTTTGATGAAAAAGCCCCTAGTTTGAGATTTTTTTTCTGTCGATTCGATTCTAAAGGTTTAAAATTAGAGTTTTTACATTTGTTTGATGAAAAAGGCCTTAAATTTGAGTTTTTCCGGTTGATTTGATGAAAAAGCCCTAGAATTTGTGTTTTTTCGTCGGTTTGATTCTGAAGGCCTAAAATTTGAGTTTCTCCGGCTGTTTTGATGAAAAAGCCCTAAATTTGAGTTTCTCCGGCTGTTTTGATGAAAAAGCCCTAAATTTGAGTTTTTTCCCCGTGTTTTAGATTGTTTGGTTTTAATTCTCGAATCAGCTAATCAGGGAGTGTGAAAAGCCCTAAATTTGAGTTTTTTTCGTTGTTCTGATTGTTGTTTTTATGAATTTGCAGATGCAGATCTTTGTGAAAACTCTCACCGGAAAGACTATCACCCTAGAGGTGGAAAGTTCTGATACAATCGACAACGTTAAGGCTAAGATCCAGGATAAGGAAGGAATTCCCCCGGATCAGCAAAGGCTTATCTTCGCCGGAAAGCAGTTGGAGGACGGACGTACTCTAGCTGATTACAACATCCAGAAGGAGTCTACCCTCCATTTGGTGCTCCGTCTACGTGGAGGT

A 568 bp intron immediately 5' to the initiation codon for the first ubiquitin monomer is present in the potato *Ubi7* genomic sequence. The intron region is highlighted in dark grey. Forward and reverse TALEN recognition sites are underlined. A 17bp forward TALEN binding target and a 17bp reverse TALEN binding site was separated by a 17bp spacer.

**Fig.S4 Nucleotide sequences of forward effectors E3**

>E3 (Forward TALEN) DNA

atggctcccaaaaagaagagaaaggtagaaccaggatcacctggtggacaatcacttatggacccaatacgaagcagaacgccatcaccagctagggaacttctctctggaccacagcctgatggagttcagccaactgcagatcgaggtgtttctccgccagccggtggccctttagatggactcccagcaagaagaacaatgtcccgtaccagactcccaagtccccctgccccgtcgccagccttttcagctgactccttctctgatcttcttaggcaatttgacccttctcttttcaatacatcccttttcgattcacttcctcctttcggcgcacatcatactgaggcagccaccggcgaatgggacgaagtccaaagtggtttaagggcagctgatgctccaccaccgacgatgagagtcgctgttaccgccgcacgtcctcctagagccaagccagcccctagaagacgagctgcgcaaccctccgatgcaagccctgcagctcaagtagaccttcgaacactaggttactcccagcaacaacaagaaaaaataaagccaaaggttagatcaacagttgcacaacatcacgaagccctagtcggacacggatttacacatgctcatatcgtggctctttcacaacatcctgcagctcttggaacagtcgctgtcaaatatcaggatatgattgctgcattgccagaagctactcacgaagctatcgtcggagttgggaaacaatggtcaggcgcaagagcattagaggcgcttctcaccgtagctggtgaattacgaggtcctccactccaattggatactgggcaattattaaaaatcgctaaacgaggtggagtcactgctgtcgaagccgttcatgcatggcgtaacgctctcacgggggccccactaaaccttaccccacaacaagttgtggcaatagcttctaatggtggtggtaaacaagcccttgagacggttcaaagacttctaccagttctttgtcaggcacatggattgaccccacaacaggtcgtagcaatcgcatctaacggaggtggtaagcaagctcttgaaacggtacaaagattacttcccgtgctttgtcaagctcatggactcactcctcaacaagtggtcgctattgcaagtaacggtggtggaaagcaagcactagaaaccgtccaacgactccttcctgttctctgtcaagcacatggtttgactcctcagcaggtcgtcgcaattgcatcaaacaatggaggcaaacaagctttagaaacagtacaaagactattgcccgttctttgccaagcgcatgggttaactcccgaacaagtcgttgccattgcaagtaacggaggaggtaaacaagctctcgaaacggttcaagcacttttacccgttctctgtcaagcacatggactcacacctgaacaagtagttgctatcgcatcgcatgatggtggaaaacaagcactggaaactgtacaaagacttttgccagttttatgtcaagcgcacggtcttactcctcaacaagttgtcgccattgcctctaatggaggtggaaaacaagctcttgaaactgtccagagacttctgcccgttctatgtcaggctcatgggctaacccctcaacaggttgttgcaatcgcatctaataatggaggaaaacaagctttagaaactgtccaacgactactgcccgttctctgccaagcacacggacttacaccacaacaggttgtagctatagctagcaatggtggcggtaaacaggctttggaaacagtacagcggcttctaccagtcttatgccaagcccacgggcttactcctcaacaagttgtcgccattgcctctaatggaggtggaaaacaagctcttgaaactgtccagagacttctgcccgttctatgtcaggctcatgggcttactcctgaacaggttgtcgcaatagcttcaaacggtggcggaaaacaagctcttgaaacagtgcaacgtctccttcccgtcctctgtcaggctcacggacttacgcccgaacaagttgttgctatagcttcgaatattggtggaaaacaagctctcgaaaccgtccaaaggctcctcccagtactttgccaagcacatggattaacccctgagcaagtagttgcaattgcctcgaacaatggaggaaagcaagcattagaaactgttcagagacttttgcctgtcctgtgtcaagcccacggtcttacaccagagcaggttgtcgctatagcttctaacattggtggaaagcaagctcttgagactgtgcaacgtttgcttccagtcctctgtcaagcacacggactcactccacaacaggtggttgcaattgcttcaaatggcggtggcaaacaagcattagagactgtacagagactacttcctgttctttgtcaagcacaagggctcacccctgagcaggtagtcgctatcgcctcaaatggtggcgggaagcaggccctggagactgttcagagactactgcccgtcctatgtcaggctcacggtctaacaccacaacaagtcgtcgcaatcgctagtcatgacggaggtcgacctgctctagagtcgatagtcgcacaactatcacgacctgatcccgctcttgcagcattgacaaacgatcatttagtcgcacttgcatgtttaggaggacgaccagcacttgatgccgttaagaaaggactaccgcacgcccctgcattgattaaaagaacaaacagacgaatcccggagagaacttcacatcgtgtagccaagcaacttgtcaaaagtgaactggaggagaagaaatctgaacttcgtcataaattgaaatatgtgcctcatgaatatattgaattaattgaaattgccagaaattccactcaggatagaattcttgaaatgaaggtaatggaattttttatgaaagtttatggatatagaggtaaacatttgggtggatcaaggaaaccggacggagcaatttatactgtcggatctcctattgattacggtgtgatcgtggatactaaagcttatagcggaggttataatctgccaattggccaagcagatgaaatgcaacgatatgtcgaagaaaatcaaacacgaaacaaacatatcaaccctaatgaatggtggaaagtctatccatcttctgtaacggaatttaagtttttatttgtgagtggtcactttaaaggaaactacaaagctcagcttacacgattaaatcatatcactaattgtaatggagctgttcttagtgtagaagagcttttaattggtggagaaatgattaaagccggcacattaaccttagaggaagtgagacggaaatttaataacggcgagataaacttttga

Nucleotide sequences of forward TALEN.

**Fig.S5 Amino acid sequences of forward effectors E3**

>E3 (Forward TALEN) amino acid

MAPKKKRKVEPGSPGGQSLMDPIRSRTPSPARELLSGPQPDGVQPTADRGVSPPAGGPLDGLPARRTMSRTRLPSPPAPSPAFSADSFSDLLRQFDPSLFNTSLFDSLPPFGAHHTEAATGEWDEVQSGLRAADAPPPTMRVAVTAARPPRAKPAPRRRAAQPSDASPAAQVDLRTLGYSQQQQEKIKPKVRSTVAQHHEALVGHGFTHAHIVALSQHPAALGTVAVKYQDMIAALPEATHEAIVGVGKQWSGARALEALLTVAGELRGPPLQLDTGQLLKIAKRGGVTAVEAVHAWRNALTGAPLN

LTPQQVVAIASNGGGKQALETVQRLLPVLCQAHG

LTPQQVVAIASNGGGKQALETVQRLLPVLCQAHG

LTPQQVVAIASNGGGKQALETVQRLLPVLCQAHG

LTPQQVVAIASNNGGKQALETVQRLLPVLCQAHG

LTPEQVVAIASNGGGKQALETVQALLPVLCQAHG

LTPEQVVAIASHDGGKQALETVQRLLPVLCQAHG

LTPQQVVAIASNGGGKQALETVQRLLPVLCQAHG

LTPQQVVAIASNNGGKQALETVQRLLPVLCQAHG

LTPQQVVAIASNGGGKQALETVQRLLPVLCQAHG

LTPQQVVAIASNGGGKQALETVQRLLPVLCQAHG

LTPEQVVAIASNGGGKQALETVQRLLPVLCQAHG

LTPEQVVAIASNIGGKQALETVQRLLPVLCQAHG

LTPEQVVAIASNNGGKQALETVQRLLPVLCQAHG

LTPEQVVAIASNIGGKQALETVQRLLPVLCQAHG

LTPQQVVAIASNGGGKQALETVQRLLPVLCQAQG

LTPEQVVAIASNGGGKQALETVQRLLPVLCQAHG

LTPQQVVAIASHDGG

RPALESIVAQLSRPDPALAALTNDHLVALACLGGRPALDAVKKGLPHAPALIKRTNRRIPERTSHRVAKQLVKSELEEKKSELRHKLKYVPHEYIELIEIARNSTQDRILEMKVMEFFMKVYGYRGKHLGGSRKPDGAIYTVGSPIDYGVIVDTKAYSGGYNLPIGQADEMQRYVEENQTRNKHINPNEWWKVYPSSVTEFKFLFVSGHFKGNYKAQLTRLNHITNCNGAVLSVEELLIGGEMIKAGTLTLEEVRRKFNNGEINF

SV40 nuclear localization signal (red) fused to N-terminal of Hax3, followed by repeat variable diresidues (RVD) for forward target binding (with codes in red, where NG codes for T, NN for G/A, HD for C and NI for A), truncated Hax3 C-terminal and FokI catalytic domain (red).

**Fig.S6 Nucleotide sequences of reverse effectors E4**

>E4 (Reverse TALEN) DNA

atggctcccaaaaagaagagaaaggtagaaccaggatcacctggtggacaatcacttatggacccaatacgaagcagaacgccatcaccagctagggaacttctctctggaccacagcctgatggagttcagccaactgcagatcgaggtgtttctccgccagccggtggccctttagatggactcccagcaagaagaacaatgtcccgtaccagactcccaagtccccctgccccgtcgccagccttttcagctgactccttctctgatcttcttaggcaatttgacccttctcttttcaatacatcccttttcgattcacttcctcctttcggcgcacatcatactgaggcagccaccggcgaatgggacgaagtccaaagtggtttaagggcagctgatgctccaccaccgacgatgagagtcgctgttaccgccgcacgtcctcctagagccaagccagcccctagaagacgagctgcgcaaccctccgatgcaagccctgcagctcaagtagaccttcgaacactaggttactcccagcaacaacaagaaaaaataaagccaaaggttagatcaacagttgcacaacatcacgaagccctagtcggacacggatttacacatgctcatatcgtggctctttcacaacatcctgcagctcttggaacagtcgctgtcaaatatcaggatatgattgctgcattgccagaagctactcacgaagctatcgtcggagttgggaaacaatggtcaggcgcaagagcattagaggcgcttctcaccgtagctggtgaattacgaggtcctccactccaattggatactgggcaattattaaaaatcgctaaacgaggtggagtcactgctgtcgaagccgttcatgcatggcgtaacgctctcacgggggccccactaaaccttaccccacaacaagttgtggcaatagcttctaatggaggtggtaaacaagcccttgagacggttcaaagacttctaccagttctttgtcaggcacatggattgaccccacaacaggtcgtagcaatcgcatctaacattggtggtaagcaagctcttgaaacggtacaaagattacttcccgtgctttgtcaagctcatggactcactcctcaacaagtggtcgctattgcaagtaatattggtggaaagcaagcactagaaaccgtccaacgactccttcctgttctctgtcaagcacatggtttgactcctcagcaggtcgtcgcaattgcatcaaataacggaggcaaacaagctttagaaacagtacaaagactattgcccgttctttgccaagcgcatgggttaactcccgaacaagtcgttgccattgcaagtaacaatggaggtaaacaagctctcgaaacggttcaagcacttttacccgttctctgtcaagcacatggactcacacctgaacaagtagttgctatcgcatcgaataatggtggaaaacaagcactggaaactgtacaaagacttttgccagttttatgtcaagcgcacggtcttactcctcaacaagttgtcgccattgcctctcatgatggtggaaaacaagctcttgaaactgtccagagacttctgcccgttctatgtcaggctcatgggctaacccctcaacaggttgttgcaatcgcatctaatggtggaggaaaacaagctttagaaactgtccaacgactactgcccgttctctgccaagcacacggacttacaccacaacaggttgtagctatagctagcaatattggcggtaaacaggctttggaaacagtacagcggcttctaccagtcttatgccaagcccacgggcttactcctcaacaagttgtcgccattgcctctaacggaggtggaaaacaagctcttgaaactgtccagagacttctgcccgttctatgtcaggctcatgggcttactcctgaacaggttgtcgcaatagcttcaaacattggcggaaaacaagctcttgaaacagtgcaacgtctccttcccgtcctctgtcaggctcacggacttacgcccgaacaagttgttgctatagcttcgaatattggtggaaaacaagctctcgaaaccgtccaaaggctcctcccagtactttgccaagcacatggattaacccctgagcaagtagttgcaattgcctcgcacgatggaggaaagcaagcattagaaactgttcagagacttttgcctgtcctgtgtcaagcccacggtcttacaccagagcaggttgtcgctatagcttctaatatcggtggaaagcaagctcttgagactgtgcaacgtttgcttccagtcctctgtcaagcacacggactcactccacaacaggtggttgcaattgcttcaaataatggtggcaaacaagcattagagactgtacagagactacttcctgttctttgtcaagcacaagggctcacccctgagcaggtagtcgctatcgcctcacacgacggcgggaagcaggccctggagactgttcagagactactgcccgtcctatgtcaggctcacggtctaacaccacaacaagtcgtcgcaatcgctagtaatattggaggtcgacctgctctagagtcgatagtcgcacaactatcacgacctgatcccgctcttgcagcattgacaaacgatcatttagtcgcacttgcatgtttaggaggacgaccagcacttgatgccgttaagaaaggactaccgcacgcccctgcattgattaaaagaacaaacagacgaatcccggagagaacttcacatcgtgtagccaagcaacttgtcaaaagtgaactggaggagaagaaatctgaacttcgtcataaattgaaatatgtgcctcatgaatatattgaattaattgaaattgccagaaattccactcaggatagaattcttgaaatgaaggtaatggaattttttatgaaagtttatggatatagaggtaaacatttgggtggatcaaggaaaccggacggagcaatttatactgtcggatctcctattgattacggtgtgatcgtggatactaaagcttatagcggaggttataatctgccaattggccaagcagatgaaatgcaacgatatgtcgaagaaaatcaaacacgaaacaaacatatcaaccctaatgaatggtggaaagtctatccatcttctgtaacggaatttaagtttttatttgtgagtggtcactttaaaggaaactacaaagctcagcttacacgattaaatcatatcactaattgtaatggagctgttcttagtgtagaagagcttttaattggtggagaaatgattaaagccggcacattaaccttagaggaagtgagacggaaatttaataacggcgagataaacttttga

Nucleotide sequences of forward TALEN.

**Fig.S7 Amino acid sequences of reverse effectors E4**

>E4 (Reverse TALEN) amino acid

MAPKKKRKVEPGSPGGQSLMDPIRSRTPSPARELLSGPQPDGVQPTADRGVSPPAGGPLDGLPARRTMSRTRLPSPPAPSPAFSADSFSDLLRQFDPSLFNTSLFDSLPPFGAHHTEAATGEWDEVQSGLRAADAPPPTMRVAVTAARPPRAKPAPRRRAAQPSDASPAAQVDLRTLGYSQQQQEKIKPKVRSTVAQHHEALVGHGFTHAHIVALSQHPAALGTVAVKYQDMIAALPEATHEAIVGVGKQWSGARALEALLTVAGELRGPPLQLDTGQLLKIAKRGGVTAVEAVHAWRNALTGAPLN

LTPQQVVAIASNGGGKQALETVQRLLPVLCQAHG

LTPQQVVAIASNIGGKQALETVQRLLPVLCQAHG

LTPQQVVAIASNIGGKQALETVQRLLPVLCQAHG

LTPQQVVAIASNNGGKQALETVQRLLPVLCQAHG

LTPEQVVAIASNNGGKQALETVQALLPVLCQAHG

LTPEQVVAIASNNGGKQALETVQRLLPVLCQAHG

LTPQQVVAIASHDGGKQALETVQRLLPVLCQAHG

LTPQQVVAIASNGGGKQALETVQRLLPVLCQAHG

LTPQQVVAIASNIGGKQALETVQRLLPVLCQAHG

LTPQQVVAIASNGGGKQALETVQRLLPVLCQAHG

LTPEQVVAIASNIGGKQALETVQRLLPVLCQAHG

LTPEQVVAIASNIGGKQALETVQRLLPVLCQAHG

LTPEQVVAIASHDGGKQALETVQRLLPVLCQAHG

LTPEQVVAIASNIGGKQALETVQRLLPVLCQAHG

LTPQQVVAIASNNGGKQALETVQRLLPVLCQAQG

LTPEQVVAIASHDGGKQALETVQRLLPVLCQAHG

LTPQQVVAIASNIGG

RPALESIVAQLSRPDPALAALTNDHLVALACLGGRPALDAVKKGLPHAPALIKRTNRRIPERTSHRVAKQLVKSELEEKKSELRHKLKYVPHEYIELIEIARNSTQDRILEMKVMEFFMKVYGYRGKHLGGSRKPDGAIYTVGSPIDYGVIVDTKAYSGGYNLPIGQADEMQRYVEENQTRNKHINPNEWWKVYPSSVTEFKFLFVSGHFKGNYKAQLTRLNHITNCNGAVLSVEELLIGGEMIKAGTLTLEEVRRKFNNGEINF

SV40 nuclear localization signal (red) fused to N-terminal of Hax3, followed by repeat variable diresidues (RVD) for reverse target binding (with codes in red, where NG codes for T, NN for G/A, HD for C and NI for A), truncated Hax3 C-terminal and FokI catalytic domain (red).

**Fig. S8 Junction sequences at the right border side**

Targeted-line-25 CGGCCATGCCATCTATAAAATGAAGCTTTCTGCACCTCATTTTTTTCATCTTCTATCTGA

Targeted-line-12 CGGCCATGCCATCTATAAAATGAAGCTTTCTGCACCTCATTTTTTTCATCTTCTATCTGA

Targeted-line-15 CGGCCATGCCATCTATAAAATGAAGCTTTCTGCACCTCATTTTCTTCATCTTCTATCTGA

Targeted-line-5 CGGCCATGCCATCTATAAAATGAAGCTTTCTGCACCTCATTTTTTTCATCTTCTATCTGA

Targeted-line-3 CGGCCATGCCATCTATAAAATGAAGCTTTCTGCACCTCATTTTTTTCATCTTCTATCTGA

Targeted-line-17 CGGCCATGCCATCTATAAAATGAAGCTTTCTGCACCTCATTTTTTTCATCTTCTATCTGA

Targeted-line-4 CGGCCATGCCATCTATAAAATGAAGCTTTCTGCACCTCATTTTTTTCATCTTCTATCTGA

Targeted-line-6 CGGCCATGCCATCTATAAAATGAAGCTTTCTGCACCTCATTTTTTTCATCTTCTATCTGA

Targeted-line-20 CGGCCATGCCATCTATAAAATGAAGCTTTCTGCACCTCATTTTTTTCATCTTCTATCTGA

Targeted-line-19 CGGCCATGCCATCTATAAAATGAAGCTTTCTGCACCTCATTTTTTTCATCTTCTATCTGA

Targeted-line-18 CGGCCATGCCATCTATAAAATGAAGCTTTCTGCACCTCATTTTTTTCATCTTCTATCTGA

Targeted-line-16 CGGCCATGCCATCTATAAAATGAAGCTTTCTGCACCTCATTTTTTTCATCTTCTATCTGA

Deduced Cggccatgccatctataaaatgaagctttctgcacctcatttttttcatcttctatctga

Targeted-line-13 CGGCCATGCCATCTATAAAATGAAGCTTTCTGCACCTCATTTTTTTCATCTTCTATCTGA

Targeted-line-7 CGGCCATGCCATCTATAAAATGAAGCTTTCTGCACCTCATTTTTTTCATCTTCTATCTGA

Targeted-line-2 CGGCCATGCCATCTATAAAATGAAGCTTTCTGCACCTCATTTTTTTCATCTTCTATCTGA

Targeted-line-10 CGGCCATGCCATCTATAAAATGAAGCTTTCTGCACCTCATTTTTTTCATCTTCTATCTGA

Targeted-line-9 CGGCCATGCCATCTATAAAATGAAGCTTTCTGCACCTCATTTTTTTCATCTTCTATCTGA

Targeted-line-11 CGGCCATGCCATCTATAAAATGAAGCTTTCTGCACCTCATTTTTTTCATCTTCTATCTGA

Targeted-line-1 CGGCCATGCCATCTATAAAATGAAGCTTTCTGCACCTCATTTTTTTCATCTTCTATCTGA

Targeted-line-8 CGGCCATGCCATCTATAAAATGAAGCTTTCTGCACCTCATTTTTTTCATCTTCTATCTGA

******************************************* ****************

Targeted-line-25 TTTCTATTATAATTTCTCTCAATTGCCTTCAAATTTCTCTTTAAGGTTAGAAATCTTCTC

Targeted-line-12 TTTCTATTATAATTTCTCTCAATTGCCTTCAAATTTCTCTTTAAGGTTAGAAATCTTCTC

Targeted-line-15 TTTCTATTATAATTTCTCTCGATTGCCTTCAAATTTCTCTTTAAGGTTAGAAATCTTCTC

Targeted-line-5 TTTCTATTATAATTTCTCTCAATTGCCTTCAAGTTTCTCTTTAAGGTTAGAAATCTTCTC

Targeted-line-3 TTTCTATTATAATTTCTCTCAATTGCCTTCAAATTTCTCTTTAAGGTTAGAAATCCTCTC

Targeted-line-17 TTTCTATTATAATTTCTCTCAATTGCCTTCAAATTTCTCTTTAAGGTTAGAAATCTTCTC

Targeted-line-4 TTTCTATTATAATTTCTCTCAATTGCCTTCAAATTTCTCTTTAAGGTTAGAAATCTTCTC

Targeted-line-6 TTTCTATTATAATTTCTCTCAATTGCCTTCAAATTTCTCTTTAAGGTTAGAAATCTTCTC

Targeted-line-20 TTTCTATTATAATTTCTCTCAATTGCCTTCAAATTTCTCTTTAAGGTTAGAAATCTTCTC

Targeted-line-19 TTTCTATTATAATTTCTCTCAATTGCCTTCAAATTTCTCTTTAAGGTTAGAAATCTTCTC

Targeted-line-18 TTTCTATTATAATTTCTCTCAATTGCCTTCAAATTTCTCTTTAAGGTTAGAAATCTTCTC

Targeted-line-16 TTTCTATTATAATTTCTCTCAATTGCCTTCAAATTTCTCTTTAAGGTTAGAAATCTTCTC

Deduced tttctattataatttctctcaattgccttcaaatttctctttaaggttagaaatcttctc

Targeted-line-13 TTTCTATTATAATTTCTCTCAATTGCCTTCAAATTTCTCTTTAAGGTTAGAAATCTTCTC

Targeted-line-7 TTTCTATTATAATTTCTCTCAATTGCCTTCAAATTTCTCTTTAAGGTTAGAAATCTTCTC

Targeted-line-2 TTTCTATTATAATTTCTCTCAATTGCCTTCAAATTTCTCTTTAAGGTTAGAAATCTTCTC

Targeted-line-10 TTTCTATTATAATTTCTCTCAATTGCCTTCAAATTTCTCTTTAAGGTTAGAAATCTTCTC

Targeted-line-9 TTTCTATTATAATTTCTCTCAATTGCCTTCAAATTTCTCTTTAAGGTTAGAAATCTTCTC

Targeted-line-11 TTTCTATTATAATTTCTCTCAATTGCCTTCAAATTTCTCTTTAAGGTTAGAAATCTTCTC

Targeted-line-1 TTTCTATTATAATTTCTCTCAATTGCCTTCAAATTTCTCTTTAAGGTTAGAAATCTTCTC

Targeted-line-8 TTTCTATTATAATTTCTCTCAATTGCCTTCAAATTTCTCTTTAAGGTTAGAAATCTTCTC

********************.***********.********************** ****

Targeted-line-25 TATTTTTGGTTTTTGTCTGTTTAGATTCTCGAATTAGCTAATCAGGTGCTGTTATAGCCC

Targeted-line-12 TATTTTTGGTTTTTGTCTGTTTAGATTCTCGAATTAGCTAATCAGGTGCTGTTATAGCCC

Targeted-line-15 TATTTTTGGTTTTTGTCTGTTTAGATTCTCGAATTAGCTAATCAGGTGCTGTTATAGCCC

Targeted-line-5 TATTTTTGGTTTTTGTCTGTTTAGATTCTCGAATTAGCTAATCAGGTGCTGTTATAGCCC

Targeted-line-3 TATTTTTGGTTTTTGTCTGTTTAGATTCTCGAATTAGCTAATCAGGTGCTGTTATAGCCC

Targeted-line-17 TATTTTTGGTTTTTGTCTGTTTAGATTCTCGAATTAGCTAATCAGGTGCTGTTATAGCCC

Targeted-line-4 TATTTTTGGTTTTTGTCTGTTTAGATTCTCGAATTAGCTAATCAGGTGCTGTTATAGCCC

Targeted-line-6 TATTTTTGGTTTTTGTCTGTTTAGATTCTCGAATTAGCTAATCAGGTGCTGTTATAGCCC

Targeted-line-20 TATTTTTGGTTTTTGTCTGTTTAGATTCTCGAATTAGCTAATCAGGTGCTGTTATAGCCC

Targeted-line-19 TATTTTTGGTTTTTGTCTGTTTAGATTCTCGAATTAGCTAATCAGGTGCTGTTATAGCCC

Targeted-line-18 TATTTTTGGTTTTTGTCTGTTTAGATTCTCGAATTAGCTAATCAGGTGCTGTTATAGCCC

Targeted-line-16 TATTTTTGGTTTTTGTCTGTTTAGATTCTCGAATTAGCTAATCAGGTGCTGTTATAGCCC

Deduced tatttttggtttttgtctgtttagattctcgaattagctaatcaggtgctgttatagccc

Targeted-line-13 TATTTTTGGTCTTTGTCTGTTTAGATTCTCGAATTAGCTAATCAGGTGCTGTTGTAGCCC

Targeted-line-7 TATTTTTGGTTTTTGTCTGTTTAGATTCTCGAATTAGCTAATCAGGTGCTGTTATAGCCC

Targeted-line-2 TATTTTTGGTTTTTGTCTGTTTAGATTCTCGAATTAGCTAATCAGGTGCTGTTATAGCCC

Targeted-line-10 TATTTTTGGTTTTTGTCTGTTTAGATTCTCGAATTAGCTAATCAGGTGCTGTTATAGCCC

Targeted-line-9 TATTTTTGGTTTTTGTCTGTTTAGATTCTCGAATTAGCTAATCAGGTGCTGTTATAGCCC

Targeted-line-11 TATTTTTGGTTTTTGTCTGTTTAGATTCTCGAATTAGCTAATCAGGTGCAGTTATAGCCC

Targeted-line-1 TATTTTTGGTTTTTGTCTGTTTAGATTCTCGAATTAGCTAATCAGGTGCTGTTATAGCCC

Targeted-line-8 TATTTTTGGTTTTTGTCTGTTTAGATTCTCGAATTAGCTAATCAGGTGCTGTTATAGCCC

********** **************************************:***.******

Targeted-line-25 TTAATTTTGAGTTTTTTTTCGGTTGTTTTGATGGAAAAGGCCTAAAATTTGAGTTTTTTT

Targeted-line-12 TTAATTTTGAGTTTTTTTTCGGTTGTTTTGATGGAAAAGGCCTAAAATTTGAGTCTTTTT

Targeted-line-15 TTAATTTTGAGTTTTTTTTCGGTTGTTTTGATGGAAAAGGCCTAAAATTTGAGTTTTTTT

Targeted-line-5 TTAATTTTGAGTTTTTTTTCGGTTGTTTTGATGGAAAAGGCCTAAAATTTGAGTTTTTTT

Targeted-line-3 TTAATTTTGAGTTTTTTTTCGGTTGTTTTGATGGAAAAGGCCTAAAATTTGAGTTTTTTT

Targeted-line-17 TTAATTTTGAGTTTTTTTTCGGTTGTTTTGATGGAAAAGGCCTAAAATTTGAGTTTTTTT

Targeted-line-4 TTAATTTTGAGTTTTTTTTCGGTTGTTTTGATGGAAAAGGCCTAAAATTTGAGTTTTTTT

Targeted-line-6 TTAATTTTGAGTTTTTTTTCGGTTGTTTTGATGGAAAAGGCCTAAAATTTGAGTTTTTTT

Targeted-line-20 TTAATTTTGAGTTTTTTTTCGGTTGTTTTGATGGAAAAGGCCTAAAATTTGAGTTTTTTT

Targeted-line-19 TTAATTTTGAGTTTTTTTTCGGTTGTTTTGATGGAAAAGGCCTAAAATTTGAGTTTTTTT

Targeted-line-18 TTAATTTTGAGTTTTTTTTCGGTTGTTTTGATGGAAAAGGCCTAAAATTTGAGTTTTTTT

Targeted-line-16 TTAATTTTGAGTTTTTTTTCGGTTGTTTTGATGGAAAAGGCCTAAAATTTGAGTTTTTTT

Deduced ttaattttgagttttttttcggttgttttgatggaaaaggcctaaaatttgagttttttt

Targeted-line-13 TTAATTTTGAGTTTTTTTTCGGTTGTTTTGATGGAAAAGGCCTAAAATTTGAGTTTTTTT

Targeted-line-7 TTAATTTTGAGTTTTTTTTCGGTTGTTTTGATGGAAAAGGCCTAAAATTTGAGTTTTTTT

Targeted-line-2 TTAATTTTGAGTTTTTTTTCGGTTGTCTTGATGGAAAAGGCCTAAAATTTGAGTTTTTTT

Targeted-line-10 TTAATTTTGAGTTTTTTTTCGGTTGTTTTGATGGAAAAGGCCTAAAATTTGAGTTTTTTT

Targeted-line-9 TTAATTTTGAGTTTTTTTTCGGTTGTTTTGATGGAAAAGGCCTAAAATTTGAGTTTTTTT

Targeted-line-11 TTAATTTTGAGTTTTTTTTCGGTTGTCTTGATGGAAAAGGCCTAAAATTTGAGTTTTTTT

Targeted-line-1 TTAATTTTGAGTTTTTTTTCGGTTGTCTTGATGGAAAAGGCCTAAAATTTGAGTTTTTTT

Targeted-line-8 TTAATTTTGAGTTTTTTTTCGGTTGTCTTGATGGAAAAGGCCTAAAATTTGAGTTTTTTT

************************** *************************** *****

Targeted-line-25 ACGTTGGTTTGATGGAAAAGGCCTACAATTGGAGTTTTCCCCGTTGTTTTGATGAAAAAG

Targeted-line-12 ACGTTGGTTTGATGGAAAAGGCCTACAATTGGAGTTTTCCCCGTTGTTTTGATGAAAAAG

Targeted-line-15 ACGTTGGTTTGATGGAAAAGGCCTACAATTGGAGTTTTCCCCGTTGTTTTGATGAAAAGG

Targeted-line-5 ACGTTGGTTTGATGGAAAAGGCCTACAATTGGAGTTTTCCCCGTTGTTTTGATGAAAAAG

Targeted-line-3 ACGTTGGTTCGATGGAAAAGGCCTACAATTGGAGTTTTCCCCGTTGTTTTGATGAAAAAG

Targeted-line-17 ACGTTGGTTTGATGGAAAAGGCCTACAATTGGAGTTTTCCCCGTTGTTTTGATGAAAAAG

Targeted-line-4 ACGTTGGTTTGATGGAAAAGGCCTACAATTGGAGTTTTCCCCGTTGTTTTGATGAAAAAG

Targeted-line-6 ACGTTGGTTTGATGGAAAAGGCCTACAATTGGAGTTTTCCCCGTTGTTTTGATGAAAAAG

Targeted-line-20 ACGTTGGTTTGATGGAAAAGGCCTACAATTGGAGTTTTCCCCGTTGTTTTGATGAAAAAG

Targeted-line-19 ACGTTGGTTTGATGGAAAAGGCCTACAATTGGAGTTTTCCCCGTTGTTTTGATGAAAAAG

Targeted-line-18 ACGTTGGTTTGATGGAAAAGGCCTACAATTGGAGTTTTCCCCGTTGTTTTGATGAAAAAG

Targeted-line-16 ACGTTGGTTTGATGGAAAAGGCCTACAATTGGAGTTTTCCCCGTTGTTTTGATGAAAAAG

Deduced acgttggtttgatggaaaaggcctacaattggagttttccccgttgttttgatgaaaaag

Targeted-line-13 ACGTTGGTTTGATGGAAAAGGCCTACAATTGGAGTTTTCCCCGTTGTCTTGATGAAAAAG

Targeted-line-7 ACGTTGGTTTGATGGAAAAGGCCTACAATTGGAGTTTTCCCCGTTGTTTTGATGAAAAAG

Targeted-line-2 ACGTTGGTTTGATGGAAAAGGCCTACAATTGGAGTTTTCCCCGTTGTTTTGATGAAAAAG

Targeted-line-10 ACGTTGGTTTGATGGAAAAGGCCTACAATTGGAGTTTTCCCCGTTGTTTTGATGAAAAAG

Targeted-line-9 ACGTTGGTTTGATGGAAAAGGCCTACAATTGGAGTTTTCCCCGTTGTTTTGATGAAAAAG

Targeted-line-11 ACGTTGGTTTGATGGAAAAGGCCTACAATTGGAGTTTTCCCCGTTGTTTTGATGAAAAAG

Targeted-line-1 ACGTTGGTTTGATGGAAAAGGCCTACAATTGGAGTTTTCCCCGTTGTTTTGATGAAAAAG

Targeted-line-8 ACGTTGGTTTGATGGAAAAGGCCTACAATTGGAGTTTTCCCCGTTGTTTTGATGAAAAAG

********* ************************************* **********.*

Targeted-line-25 CCCCTAGTTTGAGATTTTTTTTCTGTCGATTCGATTCTAAAGGTTTAAAATTAGAGTTTT

Targeted-line-12 CCCCTAGTTTGAGATTTT------------------------------------------

Targeted-line-15 -CCCTAGTTTGAGATTT-------------------------------------------

Targeted-line-5 CCCCTAGTTTGAGATTT-------------------------------------------

Targeted-line-3 CCCCTAGTTTGGGATTT-------------------------------------------

Targeted-line-17 CCCCTAGTTTGAGATTT-------------------------------------------

Targeted-line-4 CCCCTAGTTTGAGATTT-------------------------------------------

Targeted-line-6 CCCCTAGTTTGAGATTT-------------------------------------------

Targeted-line-20 CCCCTAGTTTGAGATTT-------------------------------------------

Targeted-line-19 CCCCTAGTTTGAGATTT-------------------------------------------

Targeted-line-18 CCCCTAGTTTGAGATTT-------------------------------------------

Targeted-line-16 CCCCTAGTTTGAGATTT-------------------------------------------

Deduced cccctagtttgagattt-------------------------------------------

Targeted-line-13 CCCCTAGTTTGAGATTT-------------------------------------------

Targeted-line-7 CCCCTAGTTTGAGATTT-------------------------------------------

Targeted-line-2 CCCCTAGTTTGAGATTT-------------------------------------------

Targeted-line-10 CCCCTAGTTTGAGATTT-------------------------------------------

Targeted-line-9 CCCCTAGTTTGAGATTT-------------------------------------------

Targeted-line-11 CCCCTAGTTTGAGATTT-------------------------------------------

Targeted-line-1 CCCCTAGTTTGAGATTT-------------------------------------------

Targeted-line-8 CCCCTAGTTTGAGATTT-------------------------------------------

**********.*****

Targeted-line-25 TACATTTGTTTGATGAAAAAGGCCTAAAATTTGAGTTTTTTTACGTTGGTTTGATGGAAA

Targeted-line-12 ------------------------------------------------------------

Targeted-line-15 ------------------------------------------------------------

Targeted-line-5 ------------------------------------------------------------

Targeted-line-3 ------------------------------------------------------------

Targeted-line-17 ------------------------------------------------------------

Targeted-line-4 ------------------------------------------------------------

Targeted-line-6 ------------------------------------------------------------

Targeted-line-20 ------------------------------------------------------------

Targeted-line-19 ------------------------------------------------------------

Targeted-line-18 ------------------------------------------------------------

Targeted-line-16 ------------------------------------------------------------

Deduced ------------------------------------------------------------

Targeted-line-13 ------------------------------------------------------------

Targeted-line-7 ------------------------------------------------------------

Targeted-line-2 ------------------------------------------------------------

Targeted-line-10 ------------------------------------------------------------

Targeted-line-9 ------------------------------------------------------------

Targeted-line-11 ------------------------------------------------------------

Targeted-line-1 ------------------------------------------------------------

Targeted-line-8 ------------------------------------------------------------

Targeted-line-25 AGGCCTACAATTGGAGTTTTCCCCGTTGTTTTGATGAAAAAGCCCCTAGTTTGAGATTTT

Targeted-line-12 -----------------------------------------------------------T

Targeted-line-15 -----------------------------------------------------------T

Targeted-line-5 -----------------------------------------------------------T

Targeted-line-3 -----------------------------------------------------------T

Targeted-line-17 -----------------------------------------------------------T

Targeted-line-4 -----------------------------------------------------------T

Targeted-line-6 -----------------------------------------------------------T

Targeted-line-20 -----------------------------------------------------------T

Targeted-line-19 -----------------------------------------------------------T

Targeted-line-18 -----------------------------------------------------------T

Targeted-line-16 -----------------------------------------------------------T

Deduced -----------------------------------------------------------t

Targeted-line-13 -----------------------------------------------------------T

Targeted-line-7 -----------------------------------------------------------T

Targeted-line-2 -----------------------------------------------------------T

Targeted-line-10 -----------------------------------------------------------T

Targeted-line-9 -----------------------------------------------------------T

Targeted-line-11 -----------------------------------------------------------T

Targeted-line-1 -----------------------------------------------------------T

Targeted-line-8 -----------------------------------------------------------T

*

Targeted-line-25 TTTTCTGTCGATTCGATTCTAAAGGTTTAAAATTAGAGTTTTTACATTTGTTTGATGAAA

Targeted-line-12 TTTTCTGTCGATTCGATTCTAAAGGTTTAAAATTAGAGTTTTTACATTTGTTTGATGAAA

Targeted-line-15 TTTTCTGTCGATTCGATTCTAAAGGTTTAAAATTAGAGTTTTTACATTTGTTTGATGAAA

Targeted-line-5 TTTTCTGTCGATTCGATTCTAAAGGTTTAAAATTAGAGTTTTTACATTTGTTTGATGAAA

Targeted-line-3 TTTTCTGTCGATTCGATTCTAAAGGTTTAAAATTAGAGTTTTTACATTTGTTTGATGAAA

Targeted-line-17 TTTTCTGTCGATTCGATTCTAAAGGTTTAAAATTAGAGTTTTTACATTTGTTTGATGAAA

Targeted-line-4 TTTTCTGTCGATTCGATTCTAAAGGTTTAAAATTAGAGTTTTTACATTTGTTTGATGAAA

Targeted-line-6 TTTTCTGTCGATTCGATTCTAAAGGTTTAAAATTAGAGTTTTTACATTTGTTTGATGAAA

Targeted-line-20 TTTTCTGTCGATTCGATTCTAAAGGTTTAAAATTAGAGTTTTTACATTTGTTTGATGAAA

Targeted-line-19 TTTTCTGTCGATTCGATTCTAAAGGTTTAAAATTAGAGTTTTTACATTTGTTTGATGAAA

Targeted-line-18 TTTTCTGTCGATTCGATTCTAAAGGTTTAAAATTAGAGTTTTTACATTTGTTTGATGAAA

Targeted-line-16 TTTTCTGTCGATTCGATTCTAAAGGTTTAAAATTAGAGTTTTTACATTTGTTTGATGAAA

Deduced ttttctgtcgattcgattctaaaggtttaaaattagagtttttacatttgtttgatgaaa

Targeted-line-13 TTTTCTGTCGATTCGATTCTAAAGGTTTAAAATTAGAGTTTTTACATTTGTTTGATGAAA

Targeted-line-7 TTTTCTGTCGATTCGATTCTAAAGGTTTAAAATTAGAGTTTTTACATTTGTTTGATGAAA

Targeted-line-2 TTTTCTGTCGATTCGATTCTAAAGGTTTAAAATTAGAGTTTTTACATTTGTTTGATGAAA

Targeted-line-10 TTTTCTGTCGATTCGATCCTAAAGGTTTAAAATTAGAGTTTTTACATTTGTTTGATGAAA

Targeted-line-9 TTTTCTGTCGATTCGATTCTAAAGGTTTAAAATTAGAGTTTTTACATTTGTTTGATGAAA

Targeted-line-11 TTTTCTGTCGATTCGATTCTAAAGGTTTAAAATTAGAGTTTTTACATTTGTTTGATGAAA

Targeted-line-1 TTTTCTGTCGATTCGATTCTAAAGGTTTAAAATTAGAGTTTTTACATTTGTTTGATGAAA

Targeted-line-8 TTTTCTGTCGATTCGATTCTAAAGGTTTAAAATTAGAGTTTTTACATTTATTTGATGAAA

***************** *******************************.**********

Targeted-line-25 AAGGCCTTAAATTTGAGTTTTTCCGGTTGATTTGATGAAAAAGCCCTAGAATTTGTGTTT

Targeted-line-12 AAGGCCTTAAATTTGAGTTTTTCCGGTTGATTTGATGAAAAAGCCCTAGAATTTGTGTTT

Targeted-line-15 AAGGCCTTAAATTTGAGTTTTTCCGGTTGATTTGATGAAAAAGCCCTAGAATTTGTGTTT

Targeted-line-5 AAGGCCTTAAATTTGAGTTTTTCCGGTTGATTTGATGAAAAAGCCCTAGAATTTGTGTTT

Targeted-line-3 AAGGCCTTAAATTTGAGTTTTTCCGGTTGATTTGATGAAAAAGCCCTAGAATTTGTGTTT

Targeted-line-17 AAGGCCTTAAATTTGAGTTTTTCCGGTTGATTTGATGAAAAAGCCCTAGAATTTGTGTTT

Targeted-line-4 AAGGCCTTAAATTTGAGTTTTTCCGGTTGATTTGATGAAAAAGCCCTAGAATTTGTGTTT

Targeted-line-6 AAGGCCTTAAATTTGAGTTTTTCCGGTTGATTTGATGAAAAAGCCCTAGAATTTGTGTTT

Targeted-line-20 AAGGCCCTAAATTTGAGTTTTTCCGGTTGATTTGATGAAAAAGCCCTAGAATTTGTGTTT

Targeted-line-19 AAGGCCTTAAATTTGAGTTTTTCCGGTTGATTTGATGAAAAAGCCCTAGAATTTGTGTTT

Targeted-line-18 AAGGCCTTAAATTTGAGTTTTTCCGGTTGATTTGATGAAAAAGCCCTAGAATTTGTGTTT

Targeted-line-16 AAGGCCTTAAATTTGAGTTTTTCCGGTTGATTTGATGAAAAAGCCCTAGAATTTGTGTTT

Deduced aaggccttaaatttgagtttttccggttgatttgatgaaaaagccctagaatttgtgttt

Targeted-line-13 AAGGCCTTAAATTTGAGTTTTTCCGGTTGATTTGATGAAAAAGCCCTAGAATTTGTGTTT

Targeted-line-7 AAGGCCTTAAATTTGAGTTTTTCCGGTTGATTTGATGAAAAAGCCCTAGAATTTGTGTTT

Targeted-line-2 AAGGCCTTAAATTTGAGTTTTTCCGGTTGATTTGATGAAAAAGCCCTAGAATTTGTGTTT

Targeted-line-10 AAGGCCTTAAATTTGAGTTTTTCCGGTTGATTTGATGAAAAAGCCCTAGAATTTGTGTTT

Targeted-line-9 AAGGCCTTAAATTTGAGTTTTTCCGGTTGATTTGATGAAAAAGCCCTAGAATTTGTGTTT

Targeted-line-11 AAGGCCTTAAATTTGAGTTTTTCCGGTTGATTTGATGAAAAAGCCCTAGAATTTGTGTTT

Targeted-line-1 AAGGCCTTAAATTTGAGTTTTTCCGGTTGGTTTGATGAAAAAGCCCTAGAATTTGTGTTT

Targeted-line-8 AAGGCCTTAAATTTGAGTTTTTCCGGTTGATTTGATGAAAAAGCCCTAGAATTTGTGTTT

****** **********************.******************************

Targeted-line-25 TTTCGTCGGTTTGATTCTGAAGGCCTAAAATTTGAGTTTCTCCGGCTGTTTTGATGAAAA

Targeted-line-12 TTTCGTCGGTTTGATTCTGAAGGCCTAAAATTTGAGTTTCTCCGGCTGTTTTGATGAAAA

Targeted-line-15 TTTCGTCGGTTTGATTCTGAAGGCCTAAAATTTGAGTTTCTCCGGCTGTTTTGATGAAAA

Targeted-line-5 TTTCGTCGGTTTGATTCTGAAGGCCTAAAATTTGAGTTTCTCCGGCTGTTTTGATGAAAA

Targeted-line-3 TTTCGTCGGTTTGATTCTGAAGGCCTAAAATTTGAGTTTCTCCGGCTGTTTTGATGAAAA

Targeted-line-17 TTTCGTCGGTTTGATTCTGAAGGCCTAAAATTTGAGTTTCTCCGGCTGTTTTGATGAAAA

Targeted-line-4 TTTCGTCGGTTTGATTCTGAAGGCCTAAAATTTGAGTTTCTCCGGCTGTTTTGATGAAAA

Targeted-line-6 TTTCGTCGGTTTGATTCTGAAGGCCTAAAATTTGAGTTTCTCCGGCTGTTTTGATGAAAA

Targeted-line-20 TTTCGTCGGTTTGATTCTGAAGGCCTAAAATTTGAGTTTCTCCGGCTGTTTTGATGAAAA

Targeted-line-19 TTTCGTCGGTTTGATTCTGAAGGCCTAAAATTTGAGTTTCTCCGGCTGTTTTGATGAAAA

Targeted-line-18 TTTCGTCGGTTTGATTCTGAAGGCCTAAAATTTGAGTTTCTCCGGCTGTTTTGATGAAAA

Targeted-line-16 TTTCGTCGGTTTGATTCTGAAGGCCTAAAATTTGAGTTTCTCCGGCTGTTTTGATGAAAA

Deduced tttcgtcggtttgattctgaaggcctaaaatttgagtttctccggctgttttgatgaaaa

Targeted-line-13 TTTCGTCGGTTTGATTCTGAAGGCCTAAAATTTGAGTTTCTCCGGCTGTTTTGATGAAAA

Targeted-line-7 TTTCGTCGGTTTGATTCTGAAGGCCTAAAATTTGAGTTTCTCCGGCTGTTTTGATGAAAA

Targeted-line-2 TTTCGTCGGTTTGATTCTGAAGGCCTAAAATTTGAGTTTCTCCGGCTGTTTTGATGAAAA

Targeted-line-10 TTTCGTCGGTTTGATTCTGAAGGCCTAAAATTTGAGTTTCTCCGGCTGTTTTGATGAAAA

Targeted-line-9 TTTCGTCGGTTTGATTCTGAAGGCCTAAAATTTGAGTTTCTCCGGCTGTTTTGATGAAAA

Targeted-line-11 TTTCGTCGGTTTGATTCTGAAGGCCTAAAATTTGAGTTTCTCCGGCTGTTTTGATGAAAA

Targeted-line-1 TTTCGTCGGTTTGATTCTGAAGGCCTAAAATTTGAGTTTCTCCGGCTGTTTTGATGAAAA

Targeted-line-8 TTTCGTCGGTTTGATTCTGAAGGCCTAAAATTTGAGTTTCTCCGGCTGTTTTGATGAAAA

************************************************************

Targeted-line-25 AGCCCTAAATTTGAGTTTCTCCGGCTGTTTTGATGAAAAAGCCCTAAATTTGAGTTTTTT

Targeted-line-12 AGCCCTAAATTTGAGTTTCTCCGGCTGTTTTGATGAAAAAGCCCTAAATTTGAGTTTTTT

Targeted-line-15 AGCCCTAAATTTGAGTTTCTCCGGCTGTTTTGATGAAAAAGCCCTAAATTTGAGTTTTTT

Targeted-line-5 AGCCCTAAATTTGAGTTTCTCCGGCTGTTTTGATGAAAGAGCCCTAAATTTGAGTTTTTT

Targeted-line-3 AGCCCTAAATTTGAGTTTCTCCGGCTGTTTTGATGAAAAAGCCCTAAATTTGAGTTTTTT

Targeted-line-17 AGCCCTAAATTTGAGTTTCTCCGGCTGTTTTGATGAAAAAGCCCTAAATTTGAGTTTTTT

Targeted-line-4 AGCCCTAAATTTGAGTTTCTCCGGCTGTTTTGATGAAAAAGCCCTAAATTTGAGTTTTTT

Targeted-line-6 AGCCCTAAATTTGAGTTTCTCCGGCTGTTTTGATGAAAAAGCCCTAAATTTGAGTTTTTT

Targeted-line-20 AGCCCTAAATTTGAGTTTCTCCGGCTGTTTTGATGAAAAAGCCCTAAATTTGAGTTTTTT

Targeted-line-19 AGCCCTAAATTTGAGTTTCTCCGGCTGTTTTGATGAAAAAGCCCTAAATTTGAGTTTTTT

Targeted-line-18 AGCCCTAAATTTGAGTTTCTCCGGCTGTTTTGATGAAAAAGCCCTAAATTTGAGTTTTTT

Targeted-line-16 AGCCCTAAATTTGAGTTTCTCCGGCTGTTTTGATGAAAAAGCCCTAAATTTGAGTTTTTT

Deduced agccctaaatttgagtttctccggctgttttgatgaaaaagccctaaatttgagtttttt

Targeted-line-13 AGCCCTAAATTTGAGTTTCTCCGGCTGTTTTGATGAAAAAGCCCTAAATTTGAGTTTTTT

Targeted-line-7 AGCCCTAAATTTGAGTTTCTCCGGCTGTTTTGATGAAAAAGCCCTAAATTTGAGTTTTTT

Targeted-line-2 AGCCCTAAATTTGAGTTTCTCCGGCTGTTTTGATGAAAAAGCCCTAAATTTGAGTTTTTT

Targeted-line-10 AGCCCTAAATTTGAGTTTCTCCGGCTGTTTTGATGAAAAAGCCCTAAATTTGAGTTTTTT

Targeted-line-9 AGCCCTAAATTTGAGTTTCTCCGGCTGTTTTGATGAAAAAGCCCTAAATTTGAGTTTTTT

Targeted-line-11 AGCCCTAAATTTGAGTTTCTCCGGCTGTTTTGATGAAAAAGCCCTAAATTTGAGTTTTTT

Targeted-line-1 AGCCCTAAATTTGAGTTTCTCCGGCTGTTTTGATGAAAAAGCCCTAAATTTGAGTT-TTT

Targeted-line-8 AGCCCTAAATTTGAGTTTCTCCGGCTGTTTTGATGAAAAAGCCCTAAATTTGAGTTTTTT

**************************************.***************** ***

Targeted-line-25 CCCCGTGTTTTAGATTGTTTGGTTTTAATTCTCGAATCAGCTAATCANGGAGTGTGAAAA

Targeted-line-12 CCCCGTGTTTTAGATTGTTTGGTTTTAATTCTCGAATCAGCTAATCAGGGAGTGTGAAAA

Targeted-line-15 CCCCGTGTTTTAGATTGTTTGGTTTTAATTCTCGAATCAGCTAATCAGGGAGTGTGAAAA

Targeted-line-5 CCCCGTGTTTTAGATTGTTTGGTT-TAATTCTCGAATCAGCTAATCAGGGAGTGTGAAAA

Targeted-line-3 CCCCGTGTTTTAGATTGTTTGGTTTTAATTCTCGAATCAGCTAATCAGGGAGTGTGAAAA

Targeted-line-17 CCCCGTGTTTTAGATTGTTTGGTTTTAATTCTCGAATCAGCTAATCAGGGAGTGTGAAAA

Targeted-line-4 CCCCGTGTTTTAGATTGTTTGGTTTTAATTCTCGAATCAGCTAATCAGGGAGTGTGAAAA

Targeted-line-6 CCCCGTGTTTTAGATTGTTTGGTTTTAATTCTCGAATCAGCTAATCAGGGAGTGTGAAAA

Targeted-line-20 CCCCGTGTTTTAGATTGTTTGGTTTTAATTCTCGAATCAGCTAATCAGGGAGTGTGAAAA

Targeted-line-19 CCCCGTGTTTTAGATTGTTTGGTTTTAATTCTCGAATCAGCTAATCAGGGAGTGCGAAAA

Targeted-line-18 CCCCGTGTTTTAGATTGTTTGGTTTTAATTCTCGAATCAGCTAATCAGGGAGTGTGAAAA

Targeted-line-16 CCCCGTGTTTTAGATTGTTTGGTTTTAATTCTCGAATCAGCTAATCAGGGAGTGTGAAAA

Deduced ccccgtgttttagattgtttggttttaattctcgaatcagctaatcagggagtgtgaaaa

Targeted-line-13 CCCCGTGTTTTAGATTGTTTGGTTTTAATTCTCGAATCAGCTAATCAGGGAGTGTGAAAA

Targeted-line-7 CCCCGTGTTTTAGATTGTTTGGTTTTAATTCTCGAATCAGCTAATCAGGGAGTGTGAAAA

Targeted-line-2 CCCCGTGTTTTAGATTGTTTGGTTTTAATTCCCGAATCAGCTAATCAGGGAGTGTGAAAA

Targeted-line-10 CCCCGTGTTTTAGATTGTTTGGTTTTAATTCTCGAATCAGCTAATCAGGGAGTGTGAAAA

Targeted-line-9 CCCCGTGTTTTAGCTTGTTTGGTTTTAATTCTCGAATCAGCTAATCAGGGAGTGTGAAAA

Targeted-line-11 CCCCGTGTTTTAGATTGTTTGGTTTTGATTCTCGAATCAGCTAATCAGGGAGTGTGAAAA

Targeted-line-1 CCCCGTGTTTTAGATTGTTTGGTTTTAATTCTCGAATCAGCTAATCAGGGAGTGTGAAAA

Targeted-line-8 CCCCGTGTTTTAGATTGTTTGGTTTTAATTCTCGAATCAGCTAATCAGGCAGTGTGAGAA

*************.********** *.**** *************** * **** **.**

Targeted-line-25 GCCCTAAAT-TTGAGTTTTTTTCGTTGTTCTGATTGTCGTTTTTATGAATTTGCAGATGC

Targeted-line-12 GCCCTAAATT-TGAG-TTTTTTCGTTGTTCTGATTGTTGTTTTTATGAATTTGCAGATGC

Targeted-line-15 GCCCTAAA-TTTGAGTTTTTTTCGTTGTTCTGATTGTTGTTTTTATGAATTTGCAGATGC

Targeted-line-5 GCCCTAAA-TTTGAGTTTTTTTCGTTGTTCTGATTGTTGTTTTTATGAATTTGCAGATGC

Targeted-line-3 GCCCTAAAATTTGAGTTTTTTTCGTTGTTCTGATTGTTGTTTTTATGAATTTGCAGATGC

Targeted-line-17 GCCCTAAAATTTGAGTTTTTTTCGTTGTTCTGATTGTTGTTTTTATGAATTTGCAGATGC

Targeted-line-4 GCCCTAAAATTTGAGTTTTTTTCGTTGTTCTGATTGTTGTTTTTATGAATTTGCAGATGC

Targeted-line-6 GCCCTAAAATTTGAGTTTTTTTCGTTGTTCTGATTGTTGTTTTTATGAATTTGCAGATGC

Targeted-line-20 GCCCTAA-ATTTGAGTTTTTTTCGTTGTTCTGATTGTTGTTTTTATGAATTTGCAGATGC

Targeted-line-19 GCCCTAA-ATTTGAGTTTTTTTCGTTGTTCTGATTGTTGTTTTTATGAATTTGCAGATGC

Targeted-line-18 GCCCTAAAATTTGAGTTTTTTTCGTTGTTCTGATTGTTGTTTTTATGAATTTGCAGATGC

Targeted-line-16 GCCCTAAAATTTGAGTTTTTTTCGTTGTTCTGATTGTTGTTTTTATGAATTTGCAGATGC

Deduced gccctaaaatttgagtttttttcgttgttctgattgttgtttttatgaatttgcagatgc

Targeted-line-13 GCCCTAAAATTTGAGTTTTTTTCGTTGTTCTGATTGTTGTTTTTATGAATTTGCAGATGC

Targeted-line-7 GCCCTAAAATTTGAGTTTTTTTCGTTGTTCTGATTGTTGTTTTTATGAATTTGCAGATGC

Targeted-line-2 GCCCTAAA-TTTGAGTTTTTTTCGTTGTTCTGATTGTTGTTTTTATGAATTTGCAGATGC

Targeted-line-10 GCCCTAAA-TTTGAGTTTTTTTCGTTGTTCTGATTGTTGTTTTTATGAATTTGCAGATGC

Targeted-line-9 GCCCTAAA-TTTGAGTTTTTTTCGTTGTTCTGATTGTTGTTTTTATGAATTTGCAGATGC

Targeted-line-11 GCCCTAAA-TTTGAGTTTTTTTCGTTGTTCTGATTGTTGTTTTTATGAATTTGCAGATGC

Targeted-line-1 GCCCTAAA-TTTGAGTTTTTTTCGTTGTTCTGATTGTTGTTTTTATGAATTTGCAGATGC

Targeted-line-8 GCCCTAAA-TTTGAGTTTTCTTCGTTGTTCTGATTGTTGTTTTTATGAATTTGCAGATGC

******* **** *** ***************** **********************

Targeted-line-25 AGATCTTTGTGAAAACTCTCACCGGAAAGACTATCACCCTAGAGGTGGAAAGTTCTGATA

Targeted-line-12 AGATCTTTGTGAAAACTCTCACCGGAAAGACTATCACCCTAGAGGTGGAAAGTTCTGATA

Targeted-line-15 AGATCTTTGTGAAAACTCTCACCGGAAAGACTATCACCCTAGAGGTGGAAAGTTCTGATA

Targeted-line-5 AGATCTTTGTGAAAACTCTCACCGGAAAGACTATCACCCTAGAGGTGGAAAGTTCTGATA

Targeted-line-3 AGATCTTTGTGAAAACTCTCACCGGAAAGACTATCACCCTAGAGGTGGAAAGTTCTGATA

Targeted-line-17 AGATCTTTGTGAAAACTCTCACCGGAAAGACTATCACCCTAGAGGTGGAAAGTTCTGATA

Targeted-line-4 AGATCTTTGTGAGAACTCTCACCGGAAAGACTATCACCCTAGAGGTGGAAAGTTCTGATA

Targeted-line-6 AGATCTTTGTGAAAACTCTCACCGGAAAGACTATCACCCTAGAGGTGGAAAGTTCTGATA

Targeted-line-20 AGATCTTTGTGAAAACTCTCACCGGAAAGACTATCACCCTAGAGGTGGAAAGTTCTGATA

Targeted-line-19 AGATCTTTGTGAAAACTCTCACCGGAAAGACTATCACCCTAGAGGTGGAAAGTTCTGATA

Targeted-line-18 AGATCTTTGTGAAAACTCTCACCGGAAAGACTATCACCCTAGAGGTGGAAAGTTCTGATA

Targeted-line-16 AGATCTTTGTGAAAACTCTCACCGGAAAGACTATCACCCTAGAGGTGGAAAGTTCTGATA

Deduced agatctttgtgaaaactctcaccggaaagactatcaccctagaggtggaaagttctgata

Targeted-line-13 AGATCTTTGTGAAAACTCTCACCGGAAAGACTATCACCCTAGAGGTGGAAAGTTCTGATA

Targeted-line-7 AGATCTTTGTGAAAACTCTCACCGGAAAGACTATCACCCTAGAGGTGGAAAGTTCTGATA

Targeted-line-2 AGATCTTTGTGAAAACTCTCACCGGAAAGACTATCACCCTAGAGGTGGAAAGTTCTGATA

Targeted-line-10 AGATCTTTGTGAAAACTCTCACCGGAAAGACTATCACCCTAGAGGTGGAAAGTTCTGATA

Targeted-line-9 AGATCTTTGTGAAAACTCTCACCGGAAAGACTATCACCCTAGAGGTGGAAAGTTCTGATA

Targeted-line-11 AGATCTTTGTGAAAACTCTCACCGGAAAGACTATCACCCTAGAGGTGGAAAGTTCTGATA

Targeted-line-1 AGATCTTTGTGACAACTTTCACCGGAAAGACTATCACCCTNGAGGTGGAAAGTTCTGATA

Targeted-line-8 AGATCTTTGTGAAAACTCTCACCGGAAAGACTATCACTCTAGAGGTGGAAAGTTCTGATA

************ **** ******************* ** *******************

Targeted-line-25 CAATCGACAACGTTAAGGCTAAGANCCAGGATA-AGGAAGGAATTCCCCCGGATCAGCAA

Targeted-line-12 CAATCGACAACGTTAAGGCTAAGATCCAGGATA-AGGAAGGAATTCCCCCGGATCAGCAA

Targeted-line-15 CAATCGACAACGTTAAGGCTAAGATCCAGGATA-AGGAAGGAATTCCCCCGGATCAGCAA

Targeted-line-5 CAATCGACAACGTTAAGGCTAAGATCCAGGATAAAGGAAGGAATTCCCCCGGATCAGCAA

Targeted-line-3 CAACCGACAACGTTACGGCTAAGATCCAGGATA-AGGAAGGAATTCCCCCGGATCAGCAA

Targeted-line-17 CAATCGACAACGTTAAGGCTAAGATCCAGGATA-AGGAAGGAATTCCCCCGGATCAGCAA

Targeted-line-4 CAATCGACAACGTTAAGGCTAAGATCCAGGATA-AGGAAGGAATTCCCCCGGATCAGCAA

Targeted-line-6 CAATCGACAACGTTAAGGCTAAGATCCAGGATA-AGGAAGGAATTCCCCCGGATCAGCAA

Targeted-line-20 CAATCGACAACGTTAAGGCTAAGATCCAGGATA-AGGAAGGAATTCCCCCGGATCAGCAA

Targeted-line-19 CAATCGACAACGTTAAGGCTAAGATCCAGGATA-AGGAAGGAATTCCCCCGGATCAGCAA

Targeted-line-18 CAATCGACAACGTTAAGGCTAAGATCCAGGATA-AGGAAGGAATTCCCCCGGATCAGCAA

Targeted-line-16 CAATCGACAACGTTAAGGCTAAGATCCAGGATA-AGGAAGGAATTCCCCCGGATCAGCAA

Deduced caatcgacaacgttaaggctaagatccaggata-aggaaggaattcccccggatcagcaa

Targeted-line-13 CAATCGACAACGTTAAGGCTAAGATCCAGGATA-AGGAAGGAATTCCCCCGGATCAGCAA

Targeted-line-7 CAATCGACAACGTTAAGGCTAAGATCCAGGATA-AGGAAGGAATTCCCCCGGATCAGCAA

Targeted-line-2 CAATCGACAACGTTAAGGCTAAGATCCAGGATA-AGGAAGGAATTCCCCCGGATCAGCAA

Targeted-line-10 CAATCGACAACGTTAAGGCTAAGATCCAGGATA-AGGAAGGAATTCCCCCGGATCAGCAA

Targeted-line-9 CAATCGACAACGTTAAGGCTAAGATCCAGGATA-AGGAAGGAATTCCCCCGGATCAGCAA

Targeted-line-11 CAATCGACAACGTTAAGGCTAAGATCCAGGATA-AGGAAGGAATTCCCCCGGATCAGCAA

Targeted-line-1 CAATCGACAACGTCAAGGATAAGATCCAGGATA-AGGAAGGAACTCCCCCGGATCAGCAA

Targeted-line-8 CAATCGACAACGTTAAGGCTAAGATCCAGGATA-AGGAAGGAATTCCCCCGGATCAGCAA

*** ********* *.**.*****.******** ********* ****************

Targeted-line-25 AGGCTTATCTTCGCCGGAAAGCAGTTGGAGGACGGACGTACTCTAGCTGATTACAACATC

Targeted-line-12 AGGCTTATCTTCGCCGGAAAGCAGTTGGAGGACGGACGTACTCTAGCTGATTACAACATC

Targeted-line-15 AGGCTTATCTTCGCCGGAAAGCAGTTGGAGGACGGACGTACTCTAGCTGATTACAACATC

Targeted-line-5 AGGCTTATCTTCGCCGGA-AGCAGTTGGAGGACGGACGTACTCTAGCTGATTACGACATC

Targeted-line-3 AGGCTTATCTTCGCCGGAAAGCAGTTGGAGGACGGACGTACTCTAGCTGATTACAACATC

Targeted-line-17 AGGCTTATCTTCGCCGGAAAGCAGTTGGAGGACGGACGTACTCTAGCTGATTACAACATC

Targeted-line-4 AGGCTTATCTTCGCCGGAAAGCAGTTGGAGGACGGACGTACTCTAGTTGATTACAACATC

Targeted-line-6 AGGCTTATCTTCGCCGGAAAGCAGTTGGAGGACGGACGTACTCTAGCTGATTACAACATC

Targeted-line-20 AGGCTTATCTTCGCCGGAAAGCAGTTGGAGGACGGACGTACTCTAGCTGATTACAACATC

Targeted-line-19 AGGCTTATCTTCGCCGGAAAGCAGTTGGAGGACGGACGTACTCTAGCTGATTACAACATC

Targeted-line-18 AGGCTTATCTTCGCCGGAAAGCAGTTGGAGGACGGACGTACTCTAGCTGATTACAACATC

Targeted-line-16 AGGCTTATCTTCGCCGGAAAGCAGTTGGAGGACGGACGTACTCTAGCTGATTACAACATC

Deduced aggcttatcttcgccggaaagcagttggaggacggacgtactctagctgattacaacatc

Targeted-line-13 AGGCTTATCTTCGCCGGAAAGCAGTTGGAGGACGGACGTACTCTAGCTGATTACAACATC

Targeted-line-7 AGGCTTATCTTCGCCGGAAAGCAGTTGGAGGACGGACGTACTCTAGCTGATTACAACATC

Targeted-line-2 AGGCTTATCTTCGCCGGAAAGCAGTTGGAGGACGGACGTACTCTAGCTGATTACAACATC

Targeted-line-10 AGGCTTATCTTCGCCGGAAAGCAGTTGGAGGACGGACGTACTCTAGCTGATTACAACATC

Targeted-line-9 AGGCTTATCTTCGCCGGAAAGCAGTTGGAGGACGGACGTACTCTAGCTGATTACAACATC

Targeted-line-11 AGGCTTATCTTCGCCGGAAAGCAGTTGGAGGACGGACGTACTCTAGCTGATTACAACATC

Targeted-line-1 AGGCTTATTTTCGCCGGAAAGCAGTTGGAGGACGGACGTACTC-AGCTGATTACAACATC

Targeted-line-8 AGGCTTATCTTCGCCGGAAAGCAGTTGGAGGACGGACGTACTCTAGCTGATTACAACATC

******** ********* ************************ ** *******.*****

Targeted-line-25 CAGAAGGAGTCTACCCTCCATTTGGTGCTCCGTCTACGTGGAGGTATGGCGGCTGCTGCC

Targeted-line-12 CAGAAGGAGTCTACCCTCCATTTGGTGCTCCGTCTACGTGGAGGTATGGCGGCTGCTGCC

Targeted-line-15 CAGAAGGAGTCTACCCTCCATTTGGTGCTCCGTCTACGTGGAGGTATGGCGGCTGCTGCC

Targeted-line-5 CAGAA-NNGTCTACCCTCCATTTGGNGCTCCGTCTACGTGGGGGTATGGCGGCCGCTGGG

Targeted-line-3 CAGAAGGAGTCTACCCTCCATTTGGTGCTCCGTCTACGTGGAGGTATGGCGGCTGCTGCC

Targeted-line-17 CAGAAGGAGTCTACCCTCCATTTGGTGCTCCGTCTACGTGGAGGTATGGCGGCTGCTGCC

Targeted-line-4 CAGAAGGAGTCTACCCTCCATTTGGTGCTCCGTCTACGTGGAGGTATGGCGGCTGCTGCC

Targeted-line-6 CGGAAGGAGTCTACCCTCCATTTGGTGCTCCGTCTACGTGGAGGTATGGCGGCTGCTGCC

Targeted-line-20 CGGAAGGAGTCTACCCTCCATTTGGTGCTCCGTCTACGTGGAGGTATGGCGGCTGCTGCC

Targeted-line-19 CAGAAGGAGTCTACCCTCCATTTGGTGCTCCGTCTACGTGGAGGTATGGCGGCTGCTGCC

Targeted-line-18 CAGAAGGAGTCTACCCTCCATTTGGTGCTCCGTCTACGTGGAGGTATGGCGGCTGCTGCC

Targeted-line-16 CAGAAGGAGTCTACCCTCCATTTGGTGCTCCGTCTACGTGGAGGTATGGCGGCTGCTGCC

Deduced cagaaggagtctaccctccatttggtgctccgtctacgtggaggtatggcggctgctgcc

Targeted-line-13 CAGAAGGAGTCTACCCTCCATTTGGTGCTCCGTCTACGTGGAGGTATGGCGGCTGCTGCC

Targeted-line-7 CAGAAGGAGTCTACCCTCCATTTGGTGCTCCGTCTACGTGGAGGTATGGCGGCTGCTGCC

Targeted-line-2 CAGAAGGAGTCTACCCTCCATTTGGTGCTCCGTCTGCGTGGAGGTATGGCGGCTGCTGCC

Targeted-line-10 CAGAAGGAGTCTACCCTCCATTTGGTGCTCCGTCTACGTGGAGGTATGGCGGCTGCTGCC

Targeted-line-9 CAGAAGGAGTCTACCCTCCATTTGGTGCTCCGTCTACGTGGAGGTATGGCGGCTGCTGCC

Targeted-line-11 CAGAAGGAGTCTACCCTCCATTTGGTGCTCCGTCTACGTGGAGGTATGGCGGCTGCTGCC

Targeted-line-1 CAGAAGGAGTCTACCCTCCATTTGGTGCTCCGTCTACGTGGAGGTACGGCGGCTGCTGCC

Targeted-line-8 CAGAAGGAGTCTACCCTCCATTTGGTGCTCCGTCTACGTGGAGGTATGGCGGCTGCTGCC

*.*** *****************.*********.*****.**** ****** ****

Targeted-line-25 TCACCATCTCCATGTTTCTCCAAAACCCTACCTCCATCTTCCTCCAAATCTTCCACCATT

Targeted-line-12 TCACCATCTCCATGTTTCTCCAAAACCCTACCTCCATCTTCCTCCAAATCTTCCACCATT

Targeted-line-15 TCACCATCTCCATGTTTCTCCAAAACCCTACCTCCATCTTCCTCCAAATCTTCCACCATT

Targeted-line-5 GCACCATCTCCACGTTTNTCCAAAACCCTACCTCCATCTTCCTCCAAATCTTCCACCATT

Targeted-line-3 TCACCATCTCCATGTTTCTCCAAAACCCTACCTCCATCTTCCTCCAAATCTTCCACCATT

Targeted-line-17 TCACCATCTCCATGTTTCTCCAAAACCCTACCTCCATCTTCCTCCAAATCTTCCACCATT

Targeted-line-4 TCACCATCTCCATGTTTCTCCAAAACCCTACCTCCATCTTCCTCCAAATCTTCCACCATT

Targeted-line-6 TCACCATCTCCATGTTTCTCCAAAACCCTACCTCCATCTTCCTCCAAATCTTCCACCATT

Targeted-line-20 TCACCATCTCCATGTTTCTCCAAAACCCTACCTCCATCTTCCTCCAAATCTTCCACCATT

Targeted-line-19 TCACCATCTCCATGTTTCTCCAAAACCCTACCTCCATCTTCCTCCAAATCTTCCACCATT

Targeted-line-18 TCACCATCTCCATGTTTCTCCAAAACCCTACCTCCATCTTCCTCCAAATCTTCCACCATT

Targeted-line-16 TCACCATCTCCATGTTTCTCCAAAACCCTACCTCCATCTTCCTCCAAATCTTCCACCATT

Deduced tcaccatctccatgtttctccaaaaccctacctccatcttcctccaaatcttccaccatt

Targeted-line-13 TCACCATCTCCATGTTTCTCCAAAACCCTACCTCCATCTTCCTCCAAATCTTCCACCATT

Targeted-line-7 TCACCATCTCCATGTTTCTCCAAAACCCTACCTCCATCTTCCTCCAAATCTTCCACCATT

Targeted-line-2 TCACCATCTCCATGTTTCTCCAAAACCCTACCTCCATCTTCCTCCAAATCTTCCACCATT

Targeted-line-10 TCACCATCTCCATGTTTCTCCAAAACCCTACCTCCATCTTCCTCCAAATCTTCCACCATT

Targeted-line-9 TCACCATCTCCATGTTTCTCCAAAACCCTACCTCCATCTTCCTCCAAATCTTCCACCATT

Targeted-line-11 TCACCATCTCCATGTTTCTCCAAAACCCTACCTCCATCTTCCTCCAAATCTTCCACCATT

Targeted-line-1 TCACCATCTCCATGTTTCTCCAAAACCCTACCTCCATCTTCCTCCAAATCTTCCACCATT

Targeted-line-8 TCACCATCTCCATGTTTCTCCAAAACCCTACCTCCATCTTCCTCCAAATCTTCCACCATT

*********** **** ******************************************

Targeted-line-25 CTTCCTAGATCTACCTTCCCTTTCCACAATCACCCTCAAAAAGCCTCACCCCTTCATCTC

Targeted-line-12 CTTCCTAGATCTACCTTCCCTTTCCACAATCACCCTCAAAAAGCCTCACCCCTTCATCTC

Targeted-line-15 CTTCCTAGATCTACCTTCCCTTTCCACAATCACCCTCAAAAAGCCTCACCCCTTCATCTC

Targeted-line-5 CTTCCTAGATCTACCTTCCCTTTCCACAATCACCCTCAAAAAGCCTCACCCCTTCATCTC

Targeted-line-3 CTTCCTAGATCTACCTTCCCTTTCCACAATCACCCTCAAAAAGCCTCACCCCTTCATCTC

Targeted-line-17 CTTCCTAGATCTACCTTCCCTCTCCACAATCACCCTCAAAAAGCCTCACCCCTTCATCTC

Targeted-line-4 CTTCCTAGATCTACCTTCCCTTTCCACAATCACCCTCAAAAAGCCTCACCCCTTCATCTC

Targeted-line-6 CTTCCTAGATCTACCTTCCCTTTCCACAATCACCCTCAAAAAGCCTCACCCCTTCATCTC

Targeted-line-20 CTTCCTAGATCTACCTTCCCTTTCCACAATCACCCTCAAAAAGCCTCACCCCTTCATCTC

Targeted-line-19 CTTCCTAGATCTGCCTTCCCTTTCCACAATCACCCTCAAAAGGCCTCACCCCTTCATCTC

Targeted-line-18 CTTCCTAGATCTACCTTCCCTTTCCACAATCACCCTCAAAAAGCCTCACCCCTTCATCTC

Targeted-line-16 CTTCCTAGATCTACCTTCCCTTTCCACAATCACCCTCAAAAAGCCTCACCCCTTCATCTC

Deduced cttcctagatctaccttccctttccacaatcaccctcaaaaagcctcaccccttcatctc

Targeted-line-13 CATCCTAGATCTACCTTCCCTTTCCACAATCACCCTCAAAAAGCCTCACCCCTTCATCTC

Targeted-line-7 CTTCCTAGATCTACCTTCCCTTTCCACAATCACCCTCAAAAAGCCTCACCCCTTCATCTC

Targeted-line-2 CTTCCTAGATCTACCTTCCCTTTCCACAATCACCCTCAAAAAGCCTCACCCCTTCATCTC

Targeted-line-10 CTTCCTAGATCTACCTTCCCTTTCCACAATCACCCTCAAAAAGCCTCACCCCTTCATCTC

Targeted-line-9 CTTCCTAGATCTACCTTCCCTTTCCACAATCACCCTCAAAAAGCCTCACCCCTTCATCTC

Targeted-line-11 CTTCCTAGATCTACCTTCCCTTTCCACAATCACCCTCAAAAAGCCTCACCCCTTCATCTC

Targeted-line-1 CTTCCTAGATCTACCTTCCCTTTCCACAATCACCCTCAAAAAGCCTCACCCCTTCATCTC

Targeted-line-8 CTTCCTAGATCTACCTTCCCTTTCCACAATCACCCTCAAAAAGCCTCACCCCTTCATCTC

*:**********.******** *******************.******************

Targeted-line-25 ACCCACACCCATCATCATCGTCGTGGTTTCGCCGTTTCCAATGTCGTCATATCCACTACC

Targeted-line-12 ACCCACACCCATCATCATCGTCGTGGTTTCGCCGTTTCCAATGTCGTCATATCCACTACC

Targeted-line-15 ACCCACACCCATCATCATCGTCGTGGTTTCGCCGTTTCCAATGTCGTCATATCCACTACC

Targeted-line-5 ACCCACACCCATCATCATCGTCGTGGTTTCGCCGTTTCCAATGTCGTCATATCCACTACC

Targeted-line-3 ACCCACACCCATCATCATCGTCGTGGTTTCGCCGTTTCCAATGTCGTCATATCCACTACC

Targeted-line-17 ACCCACACCCATCATCATCGTCGTGGTTTCGCCGTTTCCAATGTCGTCATATCCACTACC

Targeted-line-4 ACCCACACCCATCATCATCGTCGTGGTTTCGCCGTTTCCAATGTCGTCATATCCACTACC

Targeted-line-6 ACCCACACCCATCATCATCGTCGTGGTTTCGCCGTTTCCAATGTCGTCATATCCACTACC

Targeted-line-20 ACCCACACCCATCATCATCGTCGTGGTTTCGCCGTTTCCAATATCGTCATATCCACTACC

Targeted-line-19 ACCCACACCCATCATCATCGTCGTGGTTTCGCCGTTTCCAATGTCGTCATATCCACTACC

Targeted-line-18 ACCCACACCCATCATCATCGTCGTGGTTTCGCCGTTTCCAATGTCGTCATATCCACTACC

Targeted-line-16 ACCCACACCCATCATCATCGTCGTGGTTTCGCCGTTTCCAATGTCGTCATATCCACTACC

Deduced acccacacccatcatcatcgtcgtggtttcgccgtttccaatgtcgtcatatccactacc

Targeted-line-13 ACCCACACCCATCATCATCGTCGTGGTTTCGCCGTTTCCAATGTCGTCATATCCACTACC

Targeted-line-7 ACCCACACCCATCATCATCGTCGTGGTTTCGCCGTTTCCAATGTCGTCATATCCACTACC

Targeted-line-2 ACCCACACCCATCATCATCGTCGTGGTTTCGCCGTTTCCAATGTCGTCATATCCACTACC

Targeted-line-10 ACCCACACCCATCATCATCGTCGTGGTTTCGCCGTTTCCAATGTCGTCATATCCACTACC

Targeted-line-9 ACCCACACCCATCATCATCGTCGTGGTTTCGCCGTTTCCAATGTCGTCATATCCACTACC

Targeted-line-11 ACCCACACCCATCATCATCGTCGTGGTTTCGCCGTTTCCAATGTCGTCATATCCACTACC

Targeted-line-1 ACCCACACCCATCATCATCGTCGTGGTTTCGCCGTTTCCAATGTCGTCATATCCACTACC

Targeted-line-8 ACCCACACCCATCATCATCGTCGTGGTTTCGCCGTTTCCAATGTCGTCATATCCACTACC

******************************************.*****************

Targeted-line-25 ACCCATAACGACGTTTCTGAACCTGAAACATTCGTTTCCCGT

Targeted-line-12 ACCCATAACGACGTTTCTGAACCTGAAACATTCGTTTCCCGT

Targeted-line-15 ACCCATAACGACGTTTCTGAACCTGAAACATTCGTTTCCCGT

Targeted-line-5 ACCCATAACGACGTTTCTGAACCTGAAACATTCGTTCCC-GT

Targeted-line-3 ACCCATAACGACGTTTCTGAACCTGAAACATTCGTTTCCCGT

Targeted-line-17 ACCCATAACGACGTTTCTGAACCTGAAACATTCGTTTCCCGT

Targeted-line-4 ACCCATAACGACGTTTCTGAACCTGAAACATTCGTTTCCCGT

Targeted-line-6 ACCCATAACGACGTTTCTGAACCTGAAACATTCGTTTCCCGT

Targeted-line-20 ACCCATAACGACGTTTCTGAACCTGAAACATTCGTTTCCCGT

Targeted-line-19 ACCCATAACGACGTTTCTGAACCTGAAACATTCGTTTCCCGT

Targeted-line-18 ACCCATAACGACGTTTCTGAACCTGAAACATTCGTTTCCCGT

Targeted-line-16 ACCCATAACGACGTTTCTGAACCTGAAACATTCGTTTCCCGT

Deduced acccataacgacgtttctgaacctgaaacattcgtttcccgt

Targeted-line-13 ACCCATAACGACGTTTCTGAACCTGAAACATTCGTTTCCCGT

Targeted-line-7 ACCCATAACGACGTTTCTGAACCTGAAACATTCGTTTCCCGT

Targeted-line-2 ACCCATAACGACGTTTCTGAACCTGAAACATTCGTTTCCCGT

Targeted-line-10 ACCCATAACGACGTTTCTGAACCTGAAACATTCGTTTCCCGT

Targeted-line-9 ACCCATAACGACGTTTCTGAACCTGAAACATTCGTTTCCCGT

Targeted-line-11 ACCCATAACGACGTTTCTGAACCTGAAACATTCGTTTCCCGT

Targeted-line-1 ACCCATAACGACGTTTCTGAACCTGAAACATTCGTTTCCCGT

Targeted-line-8 ACCCATAACGACGTTTCTGAACCTGAAACATTCGTTTCCCGT

************************************ ** **

Junction sequence of 20 targeted transgenic lines at the RB side. The first red highlighted position is the start nucleotide of promoter-less m*StALS* gene cassette as shown in Fig. 6. The deduced sequence shows the sequence of a *StUbi7* driven *mStALS* cassette. The red highlighted ATG is the start of *Ubi7* Monomer and the green highlighted ATG is the start of *StALS*. The forward primer HD175F (within *StUbi7* promoter) and reverse primer HD198 (within *StALS* coding) are gray highlighted. All twenty lines show the promoter-less *StALS* cassette in the construct fused to endogenous *Ubi7* promoter without additional nucleotides akin to a designed *Ubi7* promoter driven *StALS* cassette. Line 25 has an extra ~160 bp nucleotides from *StUbi7* intron region.

**Fig. S9 Southern blot on selected lines with probes from TALEN vector components**


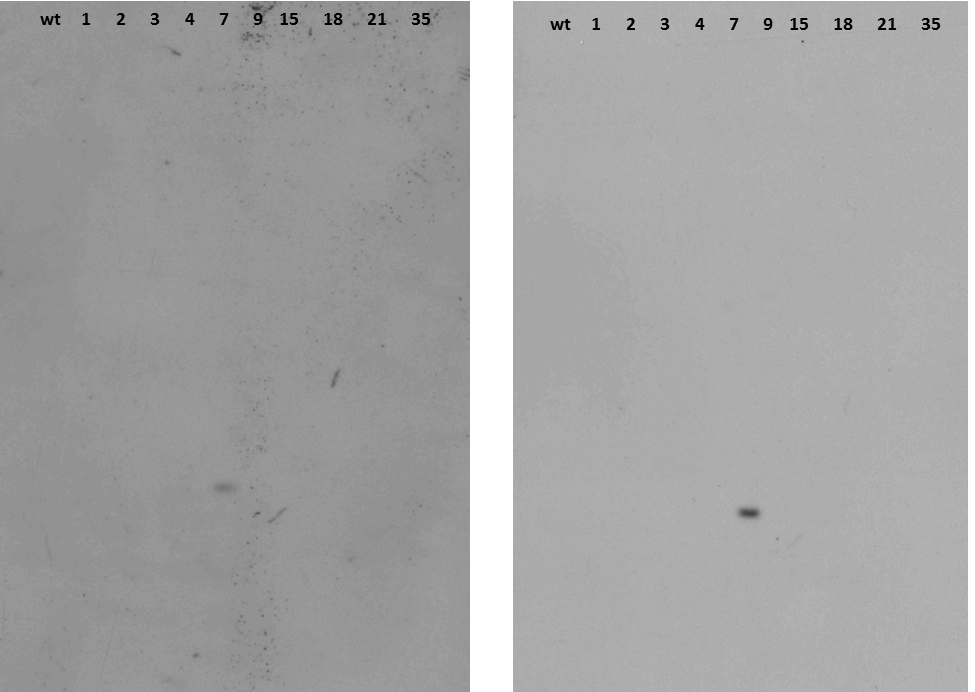


Selected lines were analyzed by Southern blot using probes targeting different region of TALEN cassette. The first blot used mixed probes targeting C terminal of designed TALEN and OCS terminator. The second blot used mixed probes targeting N terminal of designed TALEN and FMV promoter.

**Fig. S10 Expression level of transgene in random and targeted events**


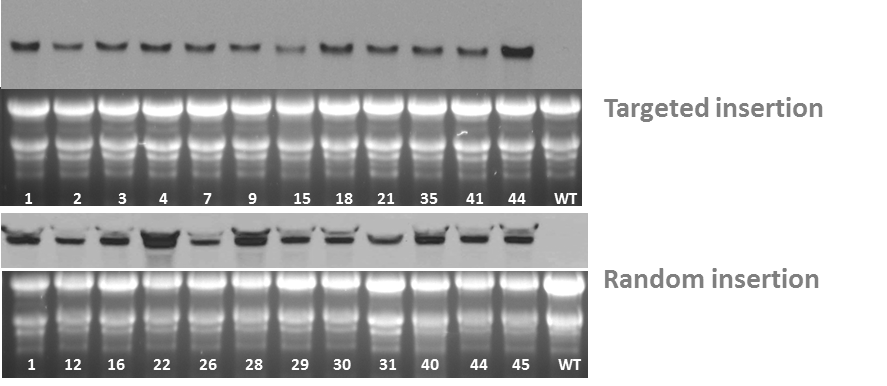


Twelve single copy lines were selected from targeted integration events and subsequently planted in the field. Leaf tissue from each line was collected and used for RNA isolation. Northern blot was performed using an *nptII* gene probe. **Top panel**: Northern blot of *nptII* gene expression in single copy targeted lines. **Bottom panel**: EB-stained ribosome RNA for same line as loading control.

**Table S1 Primers used in the method**

| **Primer name** | **Sequence** |
| --- | --- |
| HD184F | AAGGCATTCATTCCCATTTG |
| HD184R | GACCCACACTTTGCCGTAAT |
| HD175F | CGGccatgccATCTATAAAA |
| HD198 | Acgggaaacgaatgtttcag |
| HD230F | TCATCAAACCTCACCCCAAATGC |
| HD230R | TGTGATGTTCTTGTGGAGGCAC |
| HD284F | ttaaaaacgtccgcaatgtg |
| HD284R | GCGAAGATAAGCCTTTGCTG |
